# A mechanistic target of rapamycin complex 1/2 (mTORC1)/V-Akt murine thymoma viral oncogene homolog 1 (AKT1)/cathepsin H axis controls filaggrin expression and processing in skin, a novel mechanism for skin barrier disruption in patients with atopic dermatitis

**DOI:** 10.1016/j.jaci.2016.09.052

**Published:** 2017-04

**Authors:** Aishath S. Naeem, Cristina Tommasi, Christian Cole, Stuart J. Brown, Yanan Zhu, Benjamin Way, Saffron A.G. Willis Owen, Miriam Moffatt, William O. Cookson, John I. Harper, Wei-Li Di, Sara J. Brown, Thomas Reinheckel, Ryan F.L. O'Shaughnessy

**Affiliations:** a Immunobiology and Dermatology, UCL Institute of Child Health, London, United Kingdom; b Livingstone Skin Research Centre, UCL Institute of Child Health, London, United Kingdom; c Computational Biology, School of Life Sciences, University of Dundee, Dundee, United Kingdom; e Centre for Dermatology and Genetic Medicine, Medical Research Institute, University of Dundee, Dundee, United Kingdom; d National Heart and Lung Institute, Imperial College, London, United Kingdom; f Institute of Molecular Medicine and Cell Research, BIOSS Centre of Biological Signalling Studies, Albert-Ludwigs-University, Freiburg, Germany

**Keywords:** Atopic dermatitis, skin barrier, filaggrin, regulatory associated protein of the MTOR complex 1, protease, AD, Atopic dermatitis, AKT1, V-Akt murine thymoma viral oncogene homolog 1, CTSH, Cathepsin H, EM, Electron microscopy, *FLG*, Filaggrin gene, GAPDH, Glyceraldehyde-3-phosphae dehydrogenase, mTORC1/2, Mechanistic target of rapamycin complex 1/2, RAPTOR, Regulatory associated protein of the MTOR complex 1, REK, Rat epidermal keratinocyte, RXRα, Retinoid-X receptor α, shRNA, Short hairpin RNA, SNP, Single nucleotide polymorphism, WT, Wild-type

## Abstract

**Background:**

Filaggrin, which is encoded by the filaggrin gene *(FLG)*, is an important component of the skin's barrier to the external environment, and genetic defects in *FLG* strongly associate with atopic dermatitis (AD). However, not all patients with AD have *FLG* mutations.

**Objective:**

We hypothesized that these patients might possess other defects in filaggrin expression and processing contributing to barrier disruption and AD, and therefore we present novel therapeutic targets for this disease.

**Results:**

We describe the relationship between the mechanistic target of rapamycin complex 1/2 protein subunit regulatory associated protein of the MTOR complex 1 (RAPTOR), the serine/threonine kinase V-Akt murine thymoma viral oncogene homolog 1 (AKT1), and the protease cathepsin H (CTSH), for which we establish a role in filaggrin expression and processing. Increased RAPTOR levels correlated with decreased filaggrin expression in patients with AD. In keratinocyte cell cultures RAPTOR upregulation or AKT1 short hairpin RNA knockdown reduced expression of the protease CTSH. Skin of CTSH-deficient mice and CTSH short hairpin RNA knockdown keratinocytes showed reduced filaggrin processing, and the mouse had both impaired skin barrier function and a mild proinflammatory phenotype.

**Conclusion:**

Our findings highlight a novel and potentially treatable signaling axis controlling filaggrin expression and processing that is defective in patients with AD.

## Graphical abstract

**Figure:**
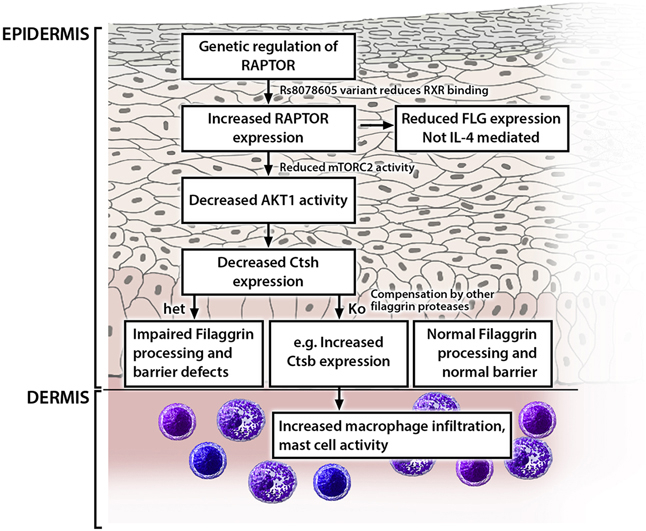

Atopic dermatitis (AD) is a common disease in which the skin is sensitive to allergens and irritants, resulting in an immune response characterized by redness and scaling. Current evidence suggests that the primary cause of disease development in the majority of patients with AD is a defective skin barrier.^1^, ^2^ There is a strong genetic component to AD associated with skin barrier dysfunction.^3^ One important protein is the epidermal structural protein filaggrin. Null mutations in the filaggrin gene *(FLG)* are responsible for the common inherited dry skin condition ichthyosis vulgaris and are a major predisposing factor for AD.^4^, ^5^ However only approximately 40% of patients with AD in the United Kingdom and around 10% of patients with AD in the rest of the world have filaggrin mutations,^6^, ^7^ and conversely, not all persons with filaggrin mutations have AD,^8^ suggesting that other mechanisms might contribute to filaggrin expression and processing defects and hence to the barrier defect observed in patients with AD.

Profilaggrin to filaggrin processing is complex, requiring dephosphorylation and numerous proteolytic events; several proteases have been identified that cleave profilaggrin at specific sites, releasing the filaggrin monomers and both the N- and C-termini.^9^ Proteases, such as elastase 2, aspartic peptidase, retroviral-like 1 (SASPase), and matriptase, are reported to be involved in profilaggrin to filaggrin processing.^10^, ^11^, ^12^, ^13^ There are also reports of aspartic- and cysteine-type cathepsin proteases playing a role in this process.^14^, ^15^, ^16^ AKT1 is required for correct formation of the cornified envelope.^18^ AKT1 activity in the epidermis is increased by treatment with the mechanistic target of rapamycin complex 1/2 (mTORC1; regulatory associated protein of the MTOR complex 1 [RAPTOR]) inhibitor rapamycin,^18^ suggesting a role of RAPTOR in modulating AKT1 activity. Therefore we hypothesized that AKT1 activity might be reduced in AD skin, leading to alteration in protease expression, reduced filaggrin expression and processing, and skin barrier disruption.

Using a combination of keratinocyte short hairpin RNA (shRNA) knockdown models, human clinical samples, and mouse knockouts, we show that increased RAPTOR expression correlates with reduced filaggrin expression in the skin of atopic subjects, being most apparent in those with *FLG* compound heterozygous mutations. RAPTOR overexpression in keratinocytes reduced filaggrin expression, loss of AKT1 activity and filaggrin, and loss of cathepsin H (CTSH). CTSH-deficient mice have reduced filaggrin processing, subtle barrier defects, and an increase in proinflammatory molecules associated with increased macrophage infiltration of the skin and increased mast cell degranulation. Taken together, this provides strong evidence that RAPTOR levels and AKT1 signaling are important in modulating filaggrin levels and the immune environment in patients with AD.

## Methods

### Animals

*Ctsh* knockout and heterozygote mice were generated, as previously described,^19^ and backcrossed onto the C57BL/6J background for eight generations. *Ctsh*^*−/−*^ and *Ctsh*^*+/−*^ mice and wild-type (WT) littermate control animals were bred under specific pathogen-free conditions in accordance with the German law for Animal Protection (Tierschutzgesetz), as published on May 25, 1998. Three-day-old (neonate) mice were obtained from 5 litters, and 6-month-old (adult) mice were obtained from 2 separate litters. A maximum of 5 WT, 8 *Ctsh*^*+/−*^, and 10 *Ctsh*^*−/−*^ neonatal mice and 3 adult mice of each phenotype were used in all analyses, and blinding was not used in the assessment of mouse skin.

### Short hairpin RNA knockdown, cell and organotypic culture, and mouse tissue

Four shRNA plasmids (Qiagen, Hilden, Germany) were used to knock down Akt1 expression (shRNA1: GCACCGCTTCTTTGCCAACAT, shRNA2: AAGGCACAGGTCGCTACTAT, shRNA3: GAGGCCCAACACCTTCATCAT, and shRNA4: GCTGTTCGAGCTCATCCTAAT), and of these, 1 and 3 were used for further experiments. Ctsh knockdown was successfully achieved by means of transient transfection with 2 shRNA plasmids (shRNA1: CAAGAATGGTCAGTGCAAATT and shRNA3: CTAGAGTCAGCTGTGGCTATT). The following scrambled control was used: GGAATCTCATTCGATGCATAC. Akt1 and Ctsh shRNA knockdown plasmids were transfected into REK cells^17^ by using lipofectamine (Invitrogen, Carlsbad, Calif), according to the manufacturer's instructions. *Mycoplasma* species testing was performed before the experiments. Cells were cultured, and G418 (Gibco, Carlsbad, Calif) selection was performed, as previously described.^17^ The organotypic cultures were either embedded in OCT for frozen sections or paraffin embedded. Drug treatments with all-trans retinoic acid (10 μmol/L; Fisher Scientific, Waltham, Mass) or wortmannin (2 μmol/L; Sigma, St Louis, Mo) were for 24 hours. Dorsal skin was removed from neonatal (postnatal day 3) *Ctsh*^*+/+*^, *Ctsh*^*+/−*^, and *Ctsh*^*−/−*^ mice for subsequent analyses.

### Lentiviral shRNA knockdown in human keratinocytes

Lentiviral particles (2 × 10^5^; scrambled control, AKT1 shRNA, and CTSH shRNA; Santa Cruz Biotechnology, Dallas, Tex) were incubated for 24 hours with 50% to 70% confluent *Mycoplasma* species–free keratinocytes grown in Gibco serum-free keratinocyte culture medium (Invitrogen) in a 12-well plate. Cells were trypsinized and selected by means of puromycin selection for 2 weeks per the manufacturer's instructions. Cells were subsequently calcium switched at 2.4 mmol/L CaCl_2_ for 4 days before investigation by using Western blotting of AKT1, CTSH, and filaggrin.

### Western blotting and antibodies

Keratinocyte protein lysates and skin protein lysates from commercially available skin samples (Caltag Medsystems, Buckingham, United Kingdom) were prepared by boiling in a denaturing SDS buffer (2% 2-mercaptoethanol, 2% SDS, and 10 mmol/L Tris [pH 7.5]) for 10 minutes. For the cytokine arrays, suspensions of T25 Ultra-Turrax (IKA, Wilmington, NC) homogenized neonatal mouse skin were spun down, and the suspensions from 2 *Ctsh*^*+/+*^, *Ctsh*^*+/−*^, and *Ctsh*^*−/−*^ mouse skin samples were pooled and used on cytokine array panel A (R&D Systems, Minneapolis, Minn), according to the manufacturers’ instructions. Densitometry of enhanced chemiluminescence exposures of cytokine arrays and Western blots, where appropriate, were performed with ImageJ software (National Institutes of Health, Bethesda, Md). Briefly, this was achieved by inverting the monochrome image, removing the background, thresholding the image, and then measuring the thresholded bands, and then the integrated density (pixel value × band area) was used as a measure of band intensity, which is subsequently normalized by using a loading control (glyceraldehyde-3-phosphae dehydrogenase [GAPDH]). Antibodies used were rabbit anti-RAPTOR (24C12; 1:500; Cell Signaling Technologies, Danvers, Mass), rabbit anti-filaggrin (M-290; no. sc-30230, 1L500; Santa Cruz Biotechnologies), mouse anti–c-Myc (9E10; 1:500; Sigma), mouse anti-FLAG (1:100; F1804, Sigma), rabbit anti-Rictor (no. 2140, 1:500; Cell Signaling Technologies), rabbit anti-pSerine473 Akt (no. 9271, 1:500; Cell Signaling Technologies), mouse anti-Akt-1 (2H10; #2967, 1:500; Cell Signaling Technologies), mouse anti-GAPDH (1:2000, AB2303; Millipore, Temecula, Calif), rabbit anti-loricrin (PRB-145P, 1:1000; Covance, Princeton, NJ), rabbit anti–keratin 10 (PRB-140C, 1:1000; Covance), rabbit anti–IL-4 (ab9622, 1:500; Abcam, Cambridge, United Kingdom), and CTSH (H-130; 1:500, sc-13988; Santa Cruz Biotechnologies). Primary antibody incubations were in PBS plus 0.1% Tween-20 or in Tris-buffered saline with 0.1% Tween 20 (100 mmol/L Tris HCl, 0.2 mol/L NaCl, and 0.1% Tween 20 [vol/vol]) containing either 5% BSA (Sigma, Gillingham, United Kingdom) or 5% skimmed milk powder either overnight at 4°C or for 1 to 2 hours at room temperature, whereas secondary antibody incubations were in 5% skimmed milk powder for 1 hour at room temperature. The following concentrations were used: swine anti-rabbit–horseradish peroxidase (1:3000; DakoCytomation, Glostrup, Denmark) and rabbit anti-mouse–horseradish peroxidase (1:2000; DakoCytomation). Protein was visualized by using the enhanced chemiluminescence plus kit (Amersham, Piscataway, NJ).

### Immunofluorescence, immunohistochemistry, and eczema and unaffected samples

Clinical material was obtained after achieving informed written consent from patients attending dermatology clinics at Great Ormond Street Hospital. Ethics approval was granted by the local research ethics committee. Normal paraffin-embedded skin samples were obtained from a commercially available tissue microarray (Biomax, Planeg, Germany), and all tissue samples were from nonflexural areas.

Immunohistochemistry and immunofluorescence on paraffin-embedded and frozen sections were done by using standard techniques. Antibodies used were RAPTOR (24C12; 1:50; Cell Signaling Technologies), mouse anti-filaggrin (GTX23137, 1/:0; GeneTex, Irvine, Calif), CTSH (H-130; sc-13988, 1:50; Santa Cruz Biotechnologies), rabbit anti-F4/80 (CL:A3:1; Bio-Rad AbD SeroTec, Hercules, Calif), rabbit anti-loricrin (PRB-145P, 1:200; Covance), rabbit anti-Il1a (H-159; sc-7929, 1:50; Santa Cruz Biotechnology), rabbit anti-cathepsin B (3190-100, 1:25; Biovision, Milpitas, Calif), rabbit anti–thymic stromal lymphopoietin (Tslp; PA5-20321, 1:25; Thermo Fisher), and rabbit anti-CD45 (EP322Y; ab40763, 1:25; Abcam). Primary antibodies were detected by using Alexa Fluor 488– and 594–conjugated goat anti-mouse and anti-rabbit (1:500; Invitrogen). Cells and sections were counterstained with 4′,6-diamidino-2-phenylindole (Sigma). Images were taken with a Leica Upright Microscope (Leica, Wetzlar, Germany) with either ×20 (NA 0.4) or ×40 (NA 1.40) objectives by using a CoolSNAP digital camera (MediaCybernetics, Bethesda, Md) with ImagePro 6.0 software (MediaCybernetics). Immunofluorescence intensity was measured with ImageJ software (https://imagej.nih.gov/ij/) to determine the integrated density on a thresholded image after processing to remove background.

### RNA extraction and microarray analysis

RNA (0.1 mg) was extracted from 2 scrambled REK lines and 2 biological replicates of each Akt1 shRNA knockdown, and poly-A+ RNA was selected by using the Oligotex system (Qiagen). RNA was extracted from the 2 Ctsh knockdown REK lines by using the same approach. Second-strand cDNA was synthesized with the Superscript II kit (Invitrogen) after the RNA was annealed with a T7 promoter-poly-T primer (Genset, Evry, France). Biotin-labeled cRNA was made from this cDNA (Enzo Diagnostics, Farmingdale, NY). The whole probe was hybridized to the Rat Exon 1.0 ST Array chip (Affymetrix, Santa Clara, Calif), according to the manufacturer's specifications. The scrambled control cells were the baseline in all analyses. Genes that were tagged as present and increased in all 6 analyses with a *P* value of less than or equal to .05 by using Mann-Whitney analysis, a *P* value of less than .05 after Benjamini-Hochberg false discovery rate correction, and 1.5-fold or more altered in expression were regarded as differentially expressed. Supervised analysis of overrepresented genes was performed by inputting lists of differentially expressed genes into the Gene Set Enrichment Analysis program (http://software.broadinstitute.org/gsea/index.jsp).

### Electron microscopy

Transmission electron microscopy (EM) was performed on WT littermates and *Ctsh* heterozygous and null mouse tissue (n = 2 each genotype). Normal EM protocols were used. Briefly, tissues were fixed overnight in glutaraldehyde, with postfixation in 1% osmium tetroxide in 100 mmol/L phosphate buffer for 2 hours at 4°C. En bloc staining with 2% aqueous uranyl acetate was performed for 2 hours before embedding and cutting of semithin sections and sections for EM grids.

### Real-time PCR

Rat CTSH and filaggrin message levels were measured by using gene-specific QuantiTect primers (Qiagen) and SYBR green (Qiagen) and ΔΔ^CT^ relative quantification.

### Sonication assay for cornified envelopes and hematoxylin dye penetration assays

Cornified envelopes were extracted from the neonatal mouse skin by boiling for 10 minutes in 50 mmol/L Tris-HCl (pH 7.5), 2% SDS, and 5 mmol/L EDTA. Cornified envelopes were pelleted by means of centrifugation and washed in cornified envelope washing buffer (10 mmol/L Tris-HCl in 0.1% SDS). After resuspension, envelopes were counted with a hemocytometer. After sonication with a probe sonicator for 5× 1-second pulses, the intact envelopes were counted and expressed as a percentage of the unsonicated total. The hematoxylin penetration assay on neonate mouse skin and subsequent sectioning has been described previously.^20^

### Correlation analysis of RAPTOR in human expression data and code availability

Skin biopsy specimens from nonlesional and nonflexural skin biopsy specimens from 26 patients with AD and 10 nonatopic control subjects of known *FLG* genotype (*FLG* WT [n = 7], *FLG* heterozygous [n = 12], and *FLG* compound heterozygous [n = 7]) were taken, the RNA was extracted, and the direct RNA sequencing reads were processed, as described previously.^21^ The mean expression for each gene was determined across the 3 *FLG* genotypes in the samples (WT, heterozygous, and compound heterozygous) and correlated to *RAPTOR*'s expression by using the Pearson method. Any genes with an *r* value close to 1 or −1 are the most likely candidates to be coregulated with *RAPTOR* under the *FLG* genotype background. Only genes with a total mean expression across the 3 genotypes of greater than 25 reads were considered (n = 9708) to avoid genes with low counts having spurious correlations.

A significance value for the correlations can be calculated. First, the *t* statistic can be determined for gene *i* as follows:

$$t_{i}=r_{i}.\sqrt{\frac{n-2}{1-r_{i}^{2}}},$$

where *r*_*i*_ is the Pearson correlation and *n* is the number of genotypes per gene (here n = 3), which determines the *df* (n - 2). Given *t*_*i*_ and the *df*, a *P* value can be calculated from the standard *t* distribution by using the *pt* function in R software (v3.1.3). *P* values are quoted unadjusted. The code for this analysis is available from GitHub (https://github.com/drchriscole/eczemaDRS). All genes with a correlation *P* value of less than .05 and a log_2_ fold change of greater than 0.5 or less than −0.5 in the WT versus compound heterozygote comparison were considered for further investigation with STRING (http://string-db.org/).

### RFLP analysis

RFLP analysis was performed on 18 skin samples. DNA was extracted by using the DNA Mini Spin Kit (Qiagen), according to the manufacturer's instructions. The rs8078605 polymorphism introduced a *Bsm*AI site into the locus. F- CACCGCATTTGCTCTTACAA and R- CCTACACATGGTCCTTCATCC (Tm 60°C) primers produced a 454-bp amplicon. The T variant after *Bsm*AI digestion produces a 203- and 251-bp product.

### Statistical analysis

For quantitative PCR and analysis of normalized data from Western blots, *t* tests or 1-way ANOVA were used. For all other analyses, nonparametric tests were performed (Kruskal-Wallis with Dunnett *post hoc* testing). Specific analyses are also identified in the figure legends.

## Results

### Increased RAPTOR expression correlated with reduced filaggrin expression in rat epidermal keratinocytes and nonlesional AD skin

**Fig E1:**
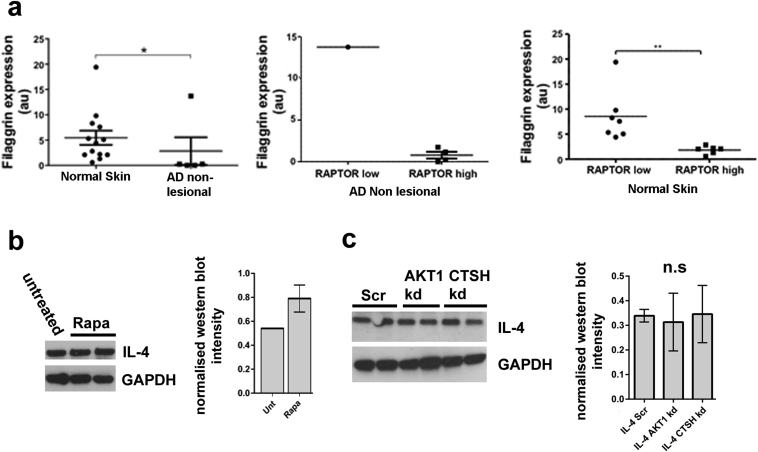


**Fig 1:**
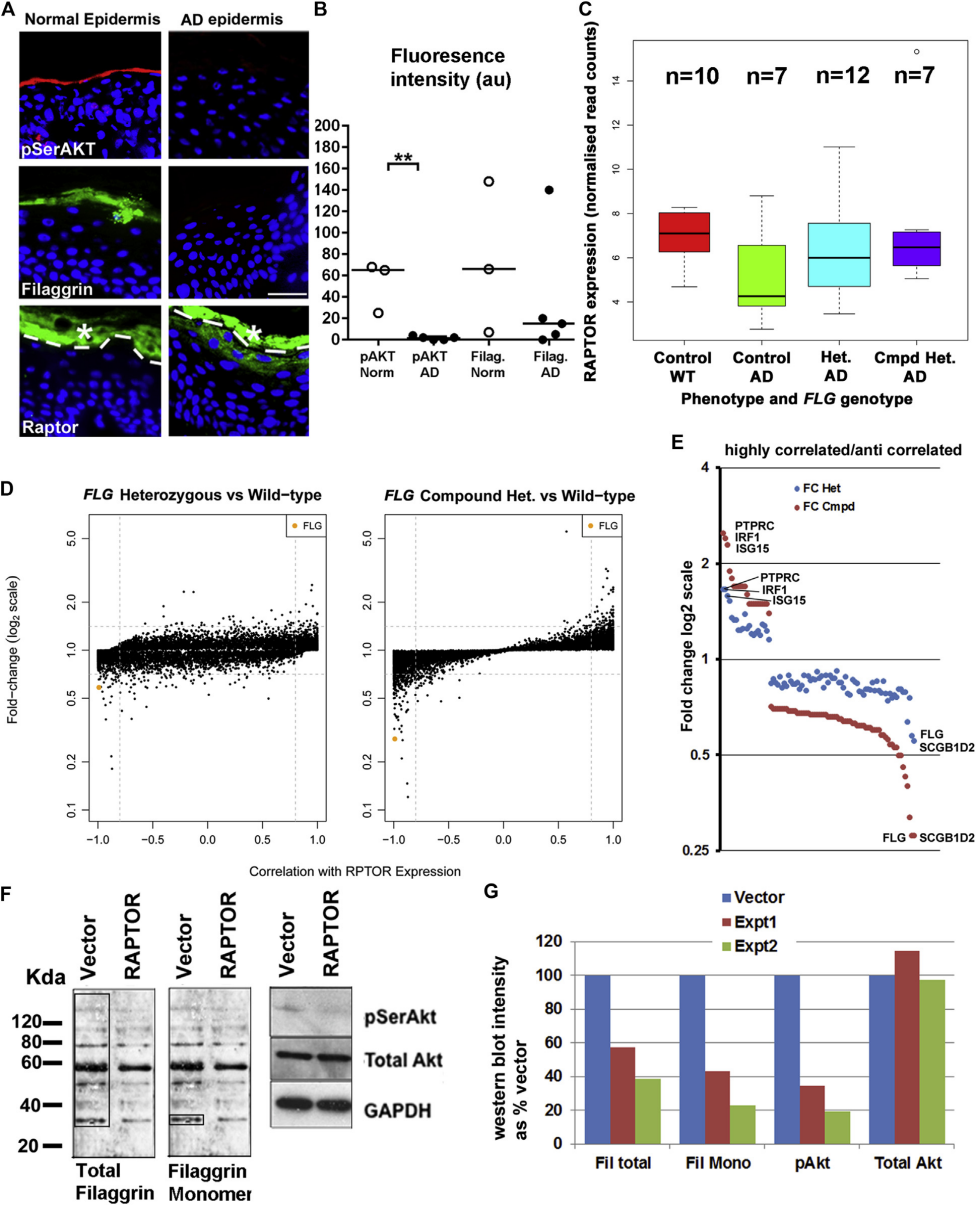
Increased RAPTOR expression correlated with reduced filaggrin expression in keratinocytes and AD skin. **A,** Filaggrin, pSerAKT, and RAPTOR immunofluorescence in normal (n = 3) and unaffected AD (n = 5) skin. **B,** Image analysis of filaggrin and pSerAKT in normal and unaffected AD skin. *Error bars* indicate SDs. **C,** RAPTOR expression from RNA sequencing analysis is described in Cole et al.^21^ The box shows medians and interquartile ranges for WT control specimens and AD specimens of the 3 *FLG* genotypes. **D,** Scatterplots showing fold change and correlation of differentially expressed genes (false discovery rate *P* < .05) with RAPTOR expression. Filaggrin (FLG) is shown in orange. **E,** Graph of fold change of highly correlated and anticorrelated genes in the *FLG* compound heterozygotes *(FC Cmpd)* and heterozygotes *(FC Het)*. **F,** Western blotting of phosphorylated Akt *(pAkt)*, total AKT, and filaggrin in RAPTOR-overexpressing keratinocytes. Boxes indicate total filaggrin and filaggrin monomer for densitometry (Fig 1, *G*). A graph of densitometry is shown in Fig 1, *F* (n = 2). GAPDH is the loading control. Fig 1, *B*: **P < .05. Fig 1, *A*: *Bars* = 50 μm.

Because inhibition of the mTORC1 complex by rapamycin increases AKT1 phosphorylation in keratinocytes,^18^ we hypothesized that the inverse could occur and that increased expression of the key mTORC1 protein RAPTOR in patients with AD would result in a reduction of AKT1 phosphorylation and therefore activity. To test this, we examined the expression of RAPTOR, phosphorylated AKT, and filaggrin in the unaffected, nonlesional, nonflexural epidermis of 5 patients with early-onset severe AD and of 3 subjects without AD (Fig 1, *A* and *B*, and Table E1 in this article's Online Repository at www.jacionline.org). Nonlesional and nonflexural skin from patients with AD has been previously demonstrated to be barrier deficient and represented a way of investigating the disease before acute immune involvement.^21^, ^22^ Phosphorylated AKT was significantly downregulated on the protein level in unaffected AD skin sections. However, in both patients with AD and control subjects, there were subjects with RAPTOR present in the spinous and granular layers, which corresponded to lower filaggrin levels in these subjects (see Fig E1, *A*, in this article's Online Repository at www.jacionline.org).

To investigate this finding in a larger number of subjects with a known *FLG* genotype, we extended our analysis of RAPTOR and filaggrin to a gene expression analysis of nonlesional and nonflexural skin biopsy specimens from 26 patients with AD and 10 nonatopic control subjects of a known *FLG* genotype, as previously described.^21^ All cases of AD had early-onset persistent and severe disease. There was no significant change in mRNA levels of RAPTOR in nonlesional atopic skin, according to the *FLG* genotype (Fig 1, *C*). However, changes in RAPTOR expression correlated with a number of the highly differentially expressed genes in *FLG* compound heterozygotes and filaggrin heterozygotes, including FLG itself (Fig 1, *D* and *E*, and see Table E2 in this article's Online Repository at www.jacionline.org). Although T_H_2 cytokines, such as IL-13 and IL-4, are known to be able to modulate expression of filaggrin and alter epidermal barrier function,^23^, ^24^ rapamycin-treated cells did not reduce IL-4 expression, and AKT1 knockdown keratinocytes did not have increased levels of IL-4 (see Fig E1, *B* and *C*). IL-4 and IL-13 expression levels were not correlated with RAPTOR levels in the data from Cole et al.^21^ Taken together, this suggests that the mechanism by which RAPTOR controls filaggrin is not due to an increase in either IL-4 or IL-13 cytokine expression.

**Fig E2:**
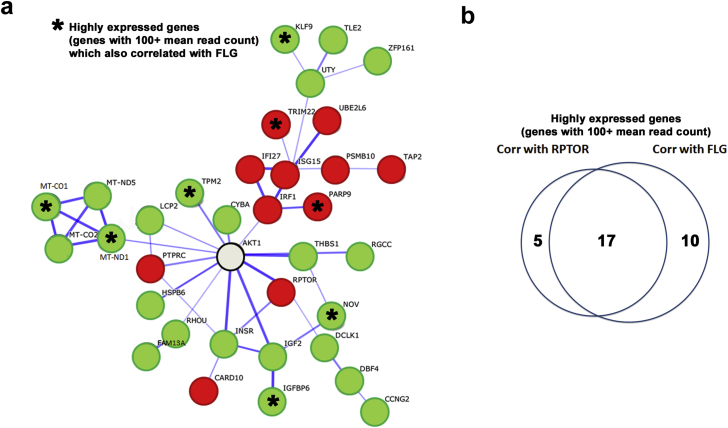


These genes and RAPTOR itself comprised a network centered on the insulin-mediated control of AKT1, which we have described previously and is important in both epidermal skin barrier function and UV protection (see Fig E2, *A*, in this article's Online Repository at www.jacionline.org).^18^ A large proportion (17/22 [77%]) of the correlated and anticorrelated highly expressed genes with a mean normalized read count of 100 or more were also genes with expression that correlated with filaggrin expression (see Fig E2, *B*, and Table E3 in this article's Online Repository at www.jacionline.org).^21^ These data demonstrate that in patients with AD, RAPTOR mRNA levels strongly anticorrelated with filaggrin mRNA expression. To directly test the effect of increased RAPTOR expression, we overexpressed human RAPTOR in rat epidermal keratinocytes (REKs). RAPTOR overexpression led to a decrease in AKT phosphorylation. Filaggrin is produced as a long proprotein, which is proteolytically processed to a monomeric mature form. We observed a reduction in total and processed monomeric filaggrin levels (Fig 1, *F* and *G*).

A single nucleotide polymorphism in RAPTOR in a retinoid-by-receptor binding site correlated with increased RAPTOR and decreased filaggrin and CTSH levels.

**Fig E3:**
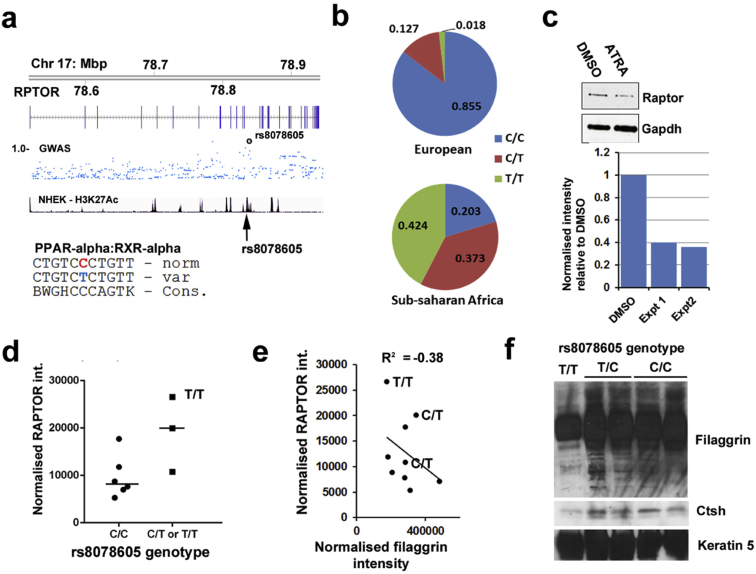


To determine whether there were genetic changes that could lead to a change in RAPTOR expression in keratinocytes, we evaluated data from a previously published genome-wide association study^25^ for any of the 649 single nucleotide polymorphisms (SNPs) in the *RAPTOR* gene that were overrepresented in patients with AD. We were not expecting gene-wide significance (*P* < 1 × 10^−8^) because RAPTOR overexpression also occurred in normal non-AD skin (see Fig E3, *A*, in this article's Online Repository at www.jacionline.org). No SNPs were significantly overrepresented, but we found an increased frequency in patients with AD of one commonly observed (>1%) SNP. rs8078605 (C>T) is in an intronic region of RAPTOR in a region of DNA, which, according to Encyclopedia of DNA Elements data,^26^ includes a region of acetylated histones in keratinocytes only, which is suggestive of a keratinocyte-specific enhancer (see Fig E3, *A*) and a binding site for retinoid-X receptor α (RXRα). The variant SNP abolished a key nucleotide of a putative RXRα-binding site. In 18 DNA samples examined 3 heterozygotes and a single homozygote were found (representing a minor allele frequency of 13.9 and 5.6% homozygotes; see Fig E3, *B*). The frequency of this variant allele in European populations was 14% compared with 79% in sub-Saharan populations. This was of particular interest because other SNPs in noncoding parts of RAPTOR with a high prevalence in sub-Saharan populations compared with European populations associated with putative retinoid binding sites that controlled the level of RAPTOR expression.^27^ Therefore we tested whether RAPTOR itself was a retinoid-responsive gene in human keratinocytes Treatment of human keratinocytes with all-trans retinoic acid reduced RAPTOR expression levels (see Fig E3, *C*), suggesting that retinoids could control RAPTOR levels in keratinocytes. Therefore we hypothesized that RXRα binding in the RAPTOR gene reduced RAPTOR expression and that the rs8078605 C>T variant would lead to increased RAPTOR expression. High-RAPTOR, low-filaggrin protein levels and low Ctsh levels correlated with the presence of the T/T variant of rs8078605 (see Fig E3, *D-F*).

### Loss of AKT1 activity or expression leads to reduced filaggrin processing in keratinocytes

**Fig 2:**
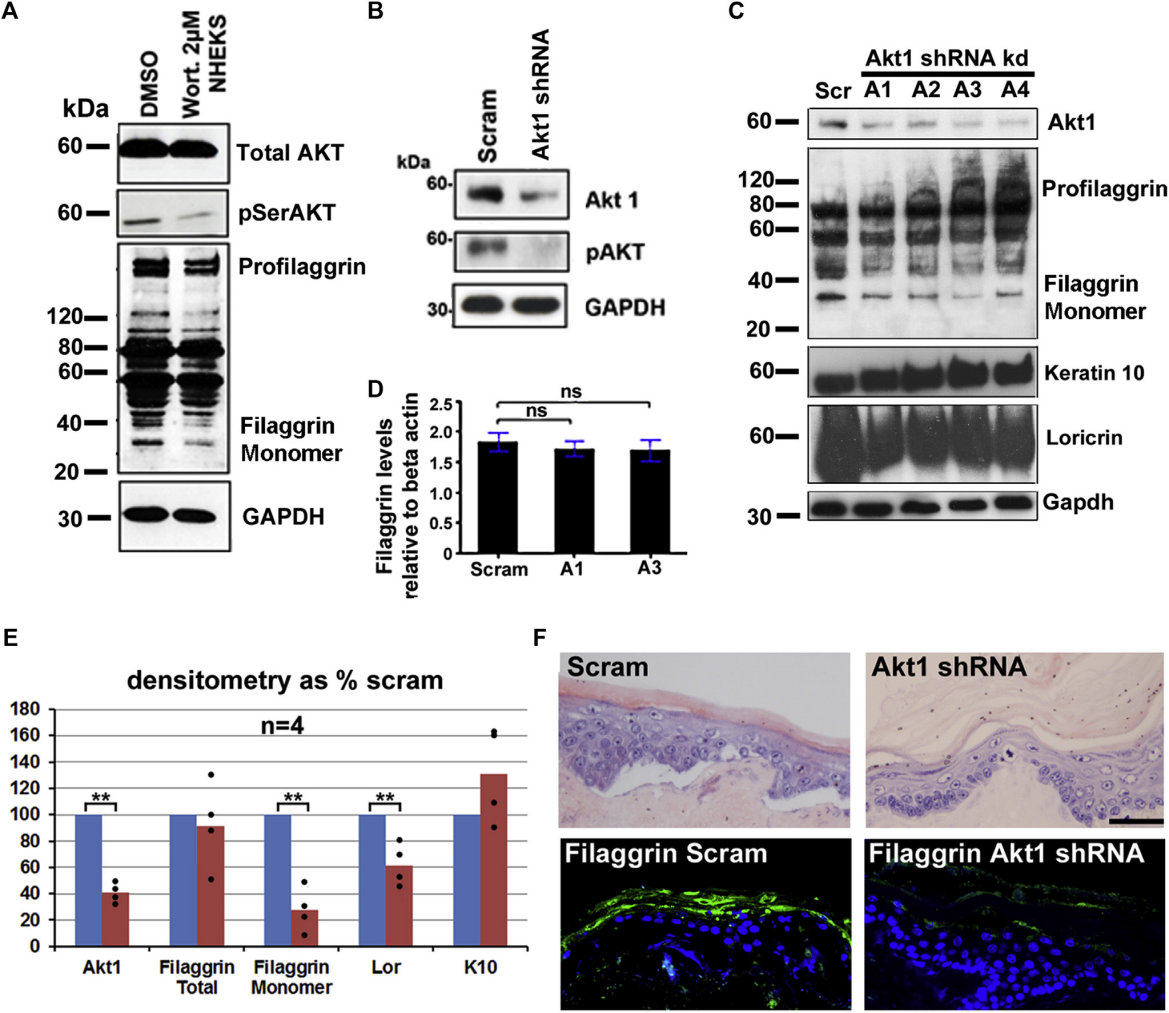
Loss of Akt1 leads to loss of filaggrin expression and hyperkeratosis in skin-equivalent organotypic cultures. **A,** Western blot of Akt, pSerAkt, and filaggrin in human keratinocytes treated with 2 μmol/L wortmannin or vehicle (dimethyl sulfoxide *[DMSO]*) for 24 hours (n = 2). **B,** Western blot of pSerAkt and Akt1 in Akt1 knockdown keratinocytes. **C,** Western blots of Akt1, filaggrin, keratin 10, and loricrin in all Akt1 shRNA–expressing lines. GAPDH is the loading control. **D,** Real-time PCR analysis of filaggrin expression in Akt1 shRNA–expressing lines. **E,** Graph of mean densitometry of Akt1, total filaggrin and filaggrin monomer, loricrin, and keratin 10 in Western blots of Akt1 shRNA. **F,** Knockdown cells *(red bars)* compared with scrambled values *(blue bars)*. Histology and immunofluorescence of Akt1 and filaggrin in Akt1 shRNA–expressing organotypic cultures (n = 4). Fig 2, *F*: *Bars* = 50 μm. **P < .05, unpaired *t* test. Error bars are SDs.

We assessed the effect of the phosphoinositide 3-kinase inhibitor wortmannin, which inhibits AKT1 phosphorylation on filaggrin expression and processing in human keratinocytes.^28^ Wortmannin treatment reduced levels of the mature processed filaggrin monomer (Fig 2, *A*). These observations suggest that phosphoinositide 3-kinase signaling through AKT1 was required for the proteolytic processing of filaggrin during late epidermal terminal differentiation. To test whether AKT1 loss was responsible for the observed changes in filaggrin expression after wortmannin treatment in keratinocytes, we transfected an REK line, which is known to represent the end stages of terminal differentiation in confluent submerged cultures,^17^ with shRNA to rat Akt1 (Fig 2, *B* and *C*). We demonstrated a significant reduction in levels of processed filaggrin monomer in 4 separate knockdown lines using Western blotting, whereas levels of total filaggrin and filaggrin mRNA remained unchanged (Fig 2, *C-E*). Organotypic skin equivalent cultures from these cells were hyperkeratotic compared with those from control cells (Fig 2, *F*). We also demonstrated a reduction in filaggrin expression in these organotypic cultures (Fig 2, *F*) with an antibody specific to the repeating mature monomeric form. These data suggest that although RAPTOR expression increase led to reduction in filaggrin expression, loss of the downstream kinase AKT1 or its activity resulted only in reduced filaggrin processing.

### mTORC signaling–related proteins and proteases, principally CTSH, are differentially expressed in Akt1 knockdown cells

**Fig E4:**
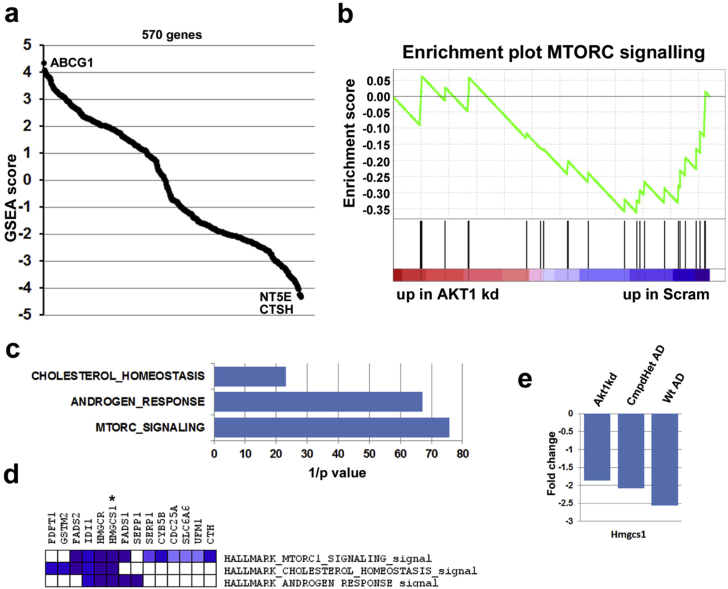


**Fig 3:**
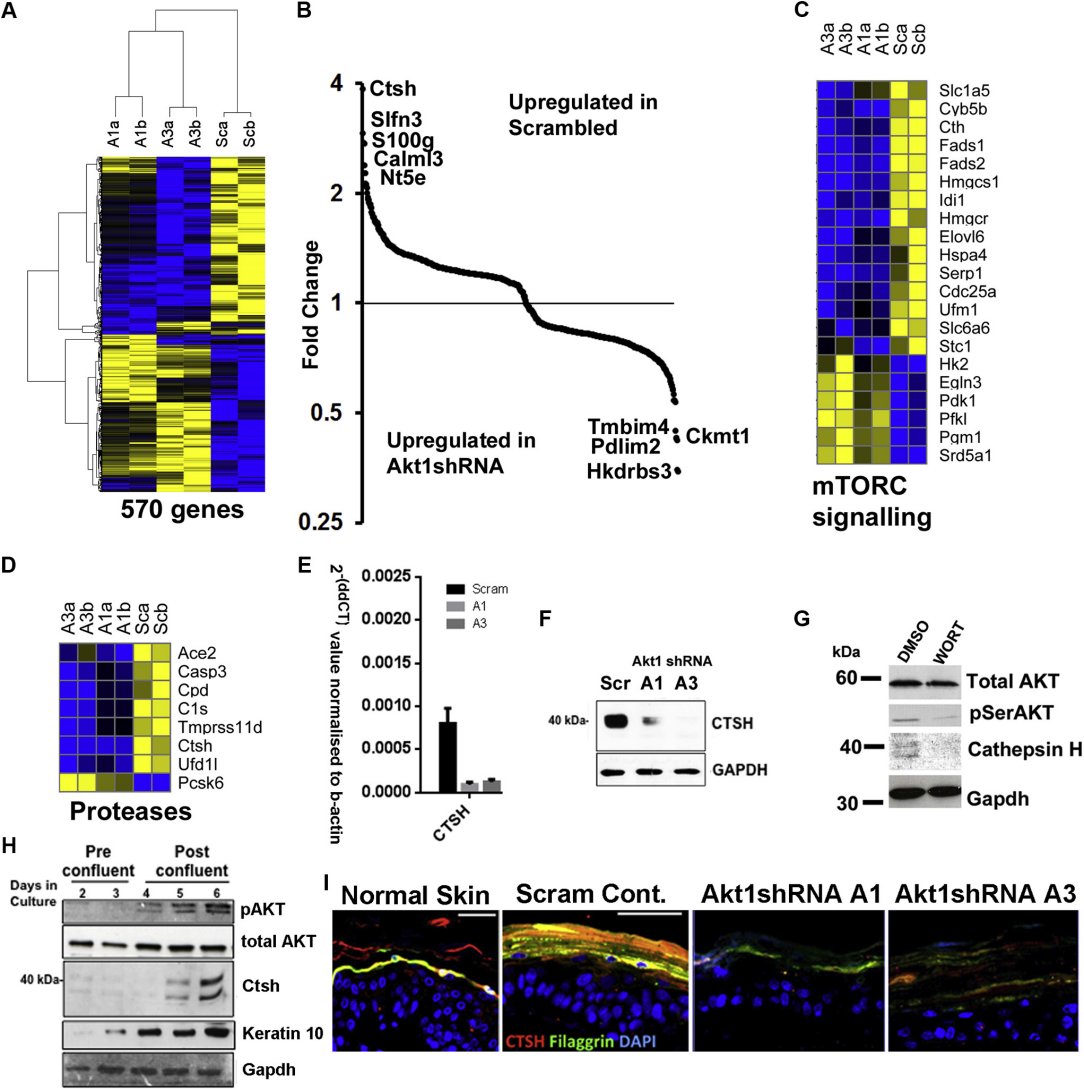
CTSH is a differentiation-dependent protease coexpressed with filaggrin. **A,** Heat map of differential gene expression between 2 Akt1 knockdown and scrambled control keratinocytes. Blue, Downregulated; yellow, upregulated. **B,** Graph of highly differentially expressed genes (DEGs), including Ctsh. **C** and **D,** Heat maps of DEGs involved in mTORC signaling (Fig 3, *C*) and proteases (Fig 3, *D*). **E,** Quantitative PCR analysis of Ctsh in Akt1 knockdown cell lines. *Bars* show SDs. *P* < .01, 2-way ANOVA. **F,** Western blot of Ctsh in Akt1 knockdown and control *(scram)* cells. **G,** Western blot of CTSH, phosphorylated AKT *(pAKT)*, and total AKT in human keratinocytes treated with wortmannin *(WORT)* or vehicle (dimethyl sulfoxide *[DMSO]*). **H,** Western blot of preconfluent and postconfluent REKs for pSerAKT, Ctsh, and keratin 1. **I,** Coimmunofluorescence of Ctsh and filaggrin. GAPDH is the loading control in all Western blots. *Bar* = 50 μm.

Differential gene expression analysis was performed on the knockdown REK lines, with the greatest reduction in Akt1 (A1 and A3). Five hundred seventy genes were significantly differentially expressed in both lines compared with scrambled controls (Fig 3, *A*). Of these, 59 genes had differential expression of 1.5-fold or greater, and 17 genes had differential expression of 2-fold or greater (Fig 3, *B*, and see Table E4 in this article's Online Repository at www.jacionline.org). Gene set enrichment analysis (see Fig E4, *A*, in this article's Online Repository at www.jacionline.org) identified 3 gene ontology groups overrepresented in the analysis, cholesterol homeostasis, androgen response, and, consistent with a role downstream of RAPTOR, MTORC signaling (Fig 3, *C*, and see Fig E3, *B* and *C*). Leading-edge analysis identified 3 genes, isopentenyl-diphosphate delta isomerase 1 *(IDI1)*, 3-hydroxy-3-methylglutaryl-CoA reductase *(HMGCR)*, and 3-hydroxy-3-methylglutaryl-CoA synthase 1 *(HMGCS1)*, in all 3 ontology groups. *HMGCS1* was downregulated in our AKT1 knockdown cells and in AD skin (see Fig E4, *D* and *E*).^21^ We identified 3 downregulated proteases or proteolysis-associated proteins in our Akt1 knockdown cell lines (Fig 3, *D*). We confirmed downregulation of the most highly downregulated of these, the lysosomal protease CTSH (3- to 4-fold), by using real-time PCR (Fig 3, *E*) and Western blotting (Fig 3, *F*). Ctsh was of particular interest because other members of the cathepsin proteases have been implicated in filaggrin processing.^14^, ^15^, ^16^ Ctsh was downregulated in human keratinocytes treated with wortmannin (Fig 3, *G*) and was expressed in postconfluent cultured REKs coincident with terminal differentiation and AKT activity (Fig 3, *H*). Reinforcing a potential role in the control of filaggrin processing, Ctsh was expressed coincident with filaggrin in the granular layer of the epidermis and organotypic cultures, with a reduction of both filaggrin and Ctsh in the Akt1 shRNA–expressing organotypic cultures (Fig 3, *I*).

### Loss of CTSH inhibits filaggrin processing, but not expression, and impairs epidermal barrier function: Evidence of compensation in CTSH knockout

**Fig E5:**
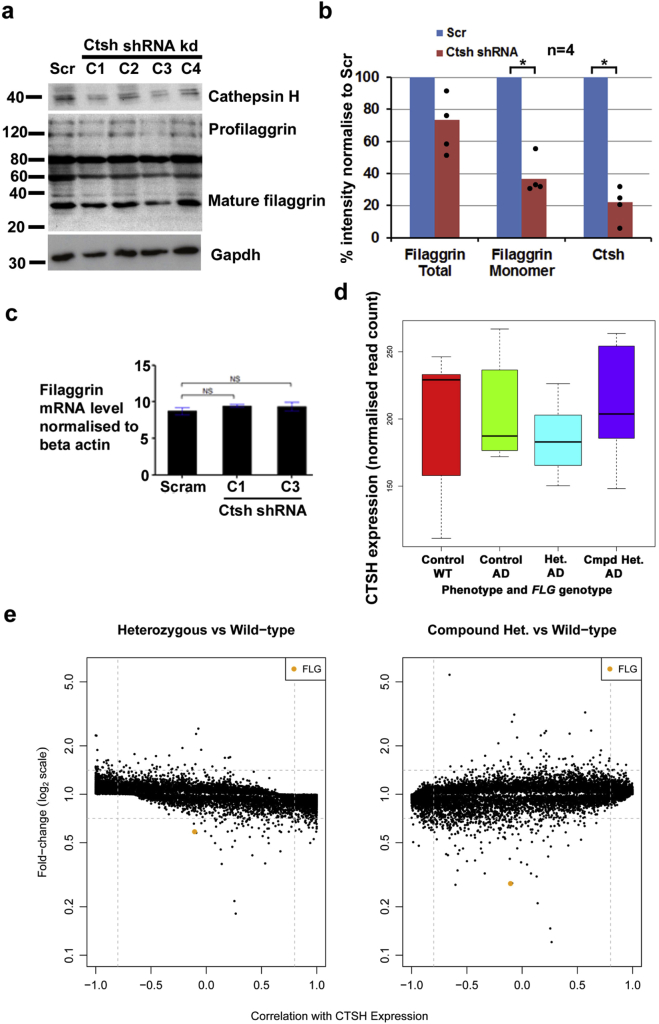


**Fig 4:**
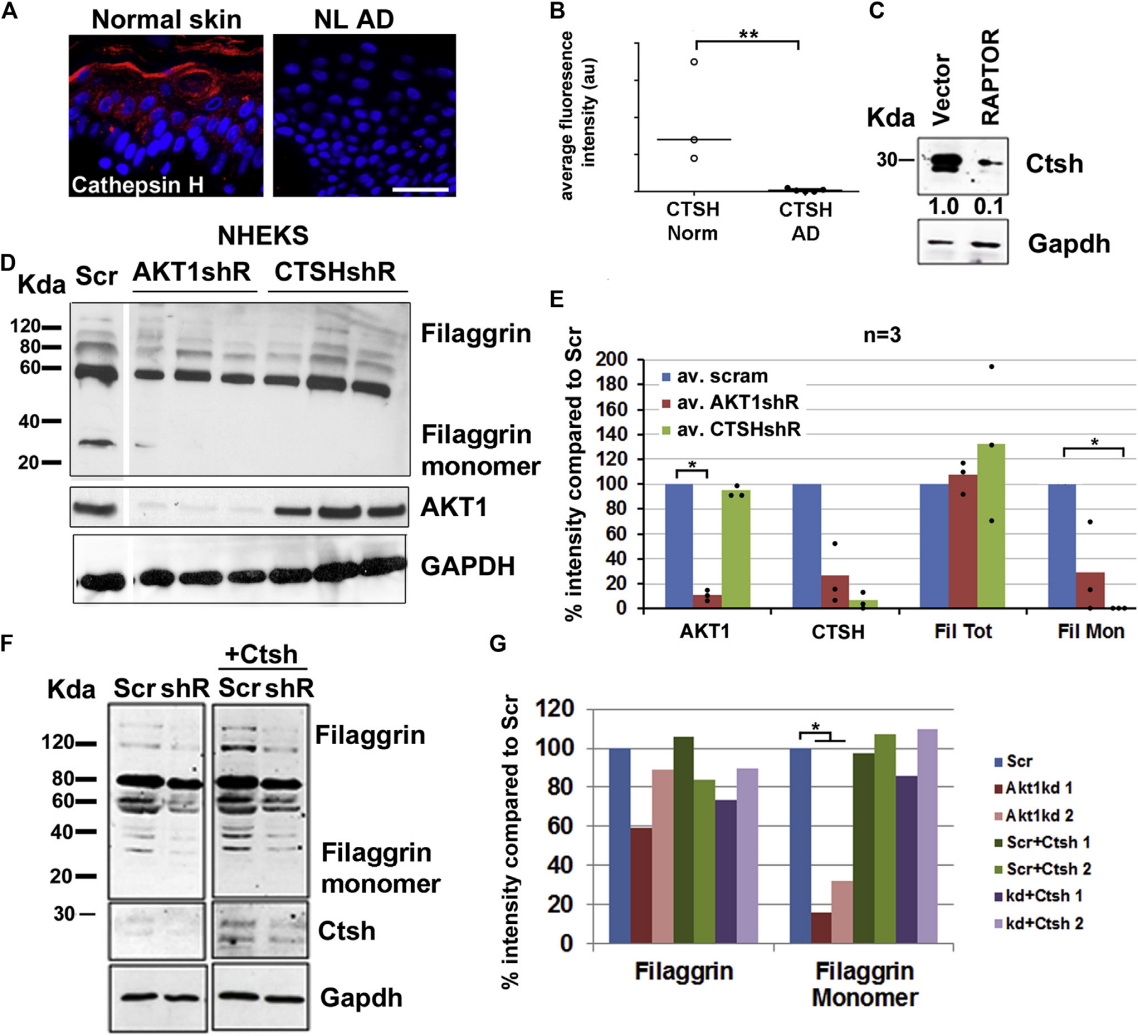
CTSH is a filaggrin-processing protease controlled by RAPTOR and AKT1. **A,** Ctsh immunofluorescence in normal and unaffected AD skin (n = 5). **B,** Graph of Ctsh fluorescence intensity. *Error bars* are SDs. **C,** Western blot of Ctsh in RAPTOR-overexpressing REKs. **D,** Western blotting for AKT1, filaggrin, and Ctsh of AKT1 and CTSH knockdown human keratinocytes *(NHEKS)*. **E,** Graph of mean densitometry of Akt1, total filaggrin and filaggrin monomer, and Ctsh. **F,** Western blot of filaggrin and Ctsh in Akt1 knockdown REKs transiently transfected with Ctsh or empty vector. **G,** Graph of mean densitometry for total filaggrin and filaggrin monomer. Two separate experiments are shown. *P < .05 and **P < .005, unpaired *t* Test (Fig 4, *E* and *G*). GAPDH is the loading control for Western blots. Fig 4, *A*: *Bar* = 50 μm.

CTSH expression was decreased in nonlesional epidermis from patients with AD (Fig 4, *A* and *B*) and was reduced in keratinocytes overexpressing RAPTOR (Fig 4, *C*), suggesting that it was a downstream effector of the RAPTOR/AKT1 axis in patients with AD. Ctsh expression was knocked down by using shRNA in our REK model to investigate a potential role for CTSH in filaggrin processing. In all 4 shRNA knockdown lines examined, there was a reduction of filaggrin processing without a reduction in filaggrin mRNA levels (see Fig E5, *A-C*, in this article's Online Repository at www.jacionline.org), which is consistent with our Akt1 knockdown data in REKs. There was a trend toward reduction of median CTSH levels in the AD RNA sequencing analysis (see Fig E5, *D*)^21^; however, consistent with the lack of change in *FLG* mRNA levels, there was no correlation between filaggrin levels and CTSH in patients with AD (see Fig E5, *E*).

Knockdown of both AKT1 and CTSH in human keratinocytes revealed the same reduction in filaggrin processing but no reduction in total filaggrin protein levels (Fig 4, *D* and *E*), strongly implying that the phenomenon we observed in the REK model was recapitulated in human keratinocytes as well, further reinforcing our finding that an increase in RAPTOR expression decreased filaggrin expression and knockdown of either AKT1 or CTSH resulted in impaired filaggrin processing only. Transient transfection of Ctsh into the Akt1 knockdown rat cell line rescued filaggrin processing (Fig 4, *F* and *G*), suggesting that loss of Ctsh was directly responsible for the reduction in filaggrin processing.

**Fig 5:**
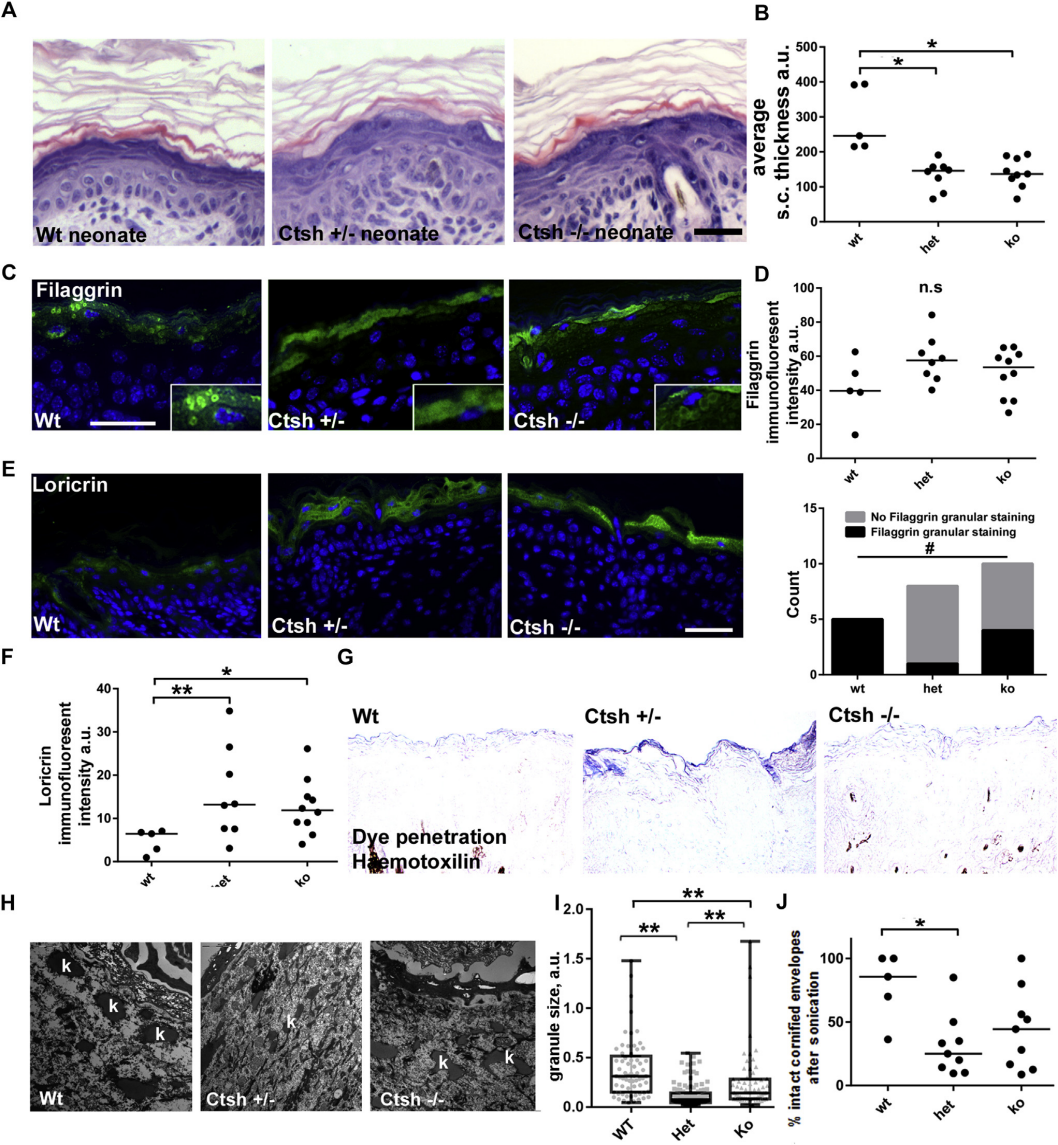
Reduced filaggrin processing and impaired epidermal barrier in CTSH-deficient mouse skin. **A,** Histology of *Ctsh*^*−/−*^, *Ctsh*^*+/−*^, and WT mouse neonatal skin (n = 5, 8, and 10, respectively). **B,** Graph of stratum corneum thickness. **C,** Filaggrin immunofluorescence. *Inset* shows granular layer detail. **D,** Graph of filaggrin immunofluorescence *(upper)* and for occurrence (counts) of granular filaggrin expression *(lower)*. **E,** Loricrin immunofluorescence. **F,** Graph of loricrin immunofluorescence intensity. **G,** Hematoxylin dye penetration. **H,** EM of keratohyalin granules. *k*, Keratohyalin granules. **I,** Graph of keratohyalin granule size. **J,** Sonication analysis of cornified envelopes. *Bars* and *boxes* shows medians and interquartile ranges (Fig 5, *I* and *J*). *P < .05, **P < .05, and #*P* < .05, Fisher exact test (Fig 5, *D*). *Bars* = 50 μm (Fig 5, *A*, *C*, and *G*) and 2 μm (Fig 5, *H*).

To investigate the effect of Ctsh reduction *in vivo*, we examined newborn mouse skin from *Ctsh*^*−/−*^ and *Ctsh*^*+/−*^ mice (Fig 5, *A* and *B*)^19^ using histology. Although there was no change in epidermal thickness, the cornified layer was significantly thinner in both the *Ctsh*^*+/−*^ and *Ctsh*^*−/−*^ mice. We observed no change in total filaggrin levels (Fig 5, *C* and *D*) but increased loricrin levels in the *Ctsh*^*+/−*^ and *Ctsh*^*−/−*^ mice using immunofluorescence (Fig 5, *E* and *F*). Granular filaggrin expression was lost in the *Ctsh*^*+/−*^ mice but was partially restored in the *Ctsh*^*−/−*^ mice (Fig 5, *D*). This was confirmed by means of Western blotting, which showed that filaggrin processing was normal and expression of loricrin and keratin 10 was increased (see Fig E7, *C*, in this article's Online Repository at www.jacionline.org). In adult mice, in contrast, expression of filaggrin and loricrin was reduced in the *Ctsh*^*+/−*^ mice and mostly restored in the *Ctsh*^*−/−*^ mouse (see Fig E7, *A*).

Dye penetration assays^20^ showed no significant gross barrier defects, but closer examination revealed penetration of dye into the cornified layers of the *Ctsh*^*+/−*^ mice (Fig 5, *G*), which is consistent with defective barrier function. EM revealed smaller keratohyalin granules specifically in both the *Ctsh*^*+/−*^ and *Ctsh*^*−/−*^ mice (Fig 5, *H* and I), but the granule size in the *Ctsh*^*−/−*^ mice was partially rescued. This was reflected in a strengthening of cornified envelope integrity in *Ctsh*^*−/−*^ mice compared with the weaker cornified envelopes in the *Ctsh*^*+/−*^ mice (Fig 5, *J*).

**Fig E6:**
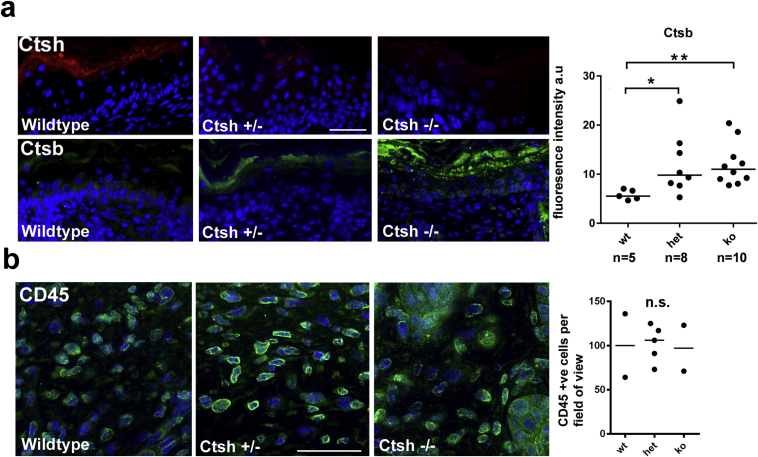


**Fig E7:**
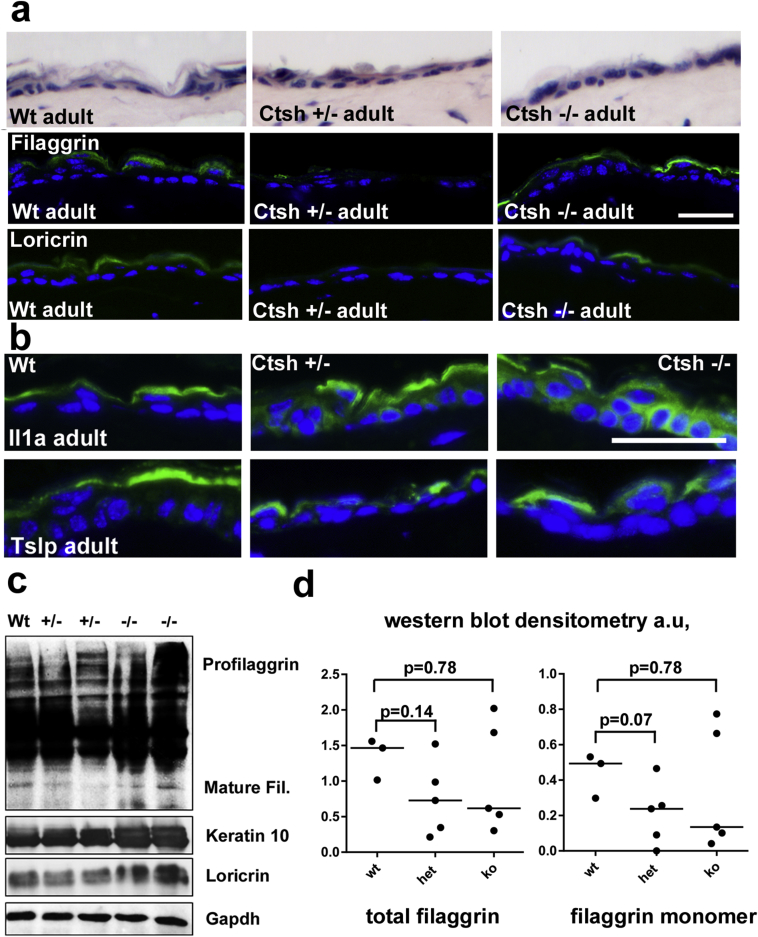


We examined the expression of other cathepsins known to process filaggrin^14^, ^15^, ^16^ to determine whether the rescue in the phenotype seen in the *Ctsh*^*−/−*^ mouse was due to some kind of compensation by another cathepsin. We were unable to detect cathepsins D and L in skin of neonates; however, the expression of cathepsin B was increased in both the knockout and heterozygous mice (see Fig E6, *A*, in this article's Online Repository at www.jacionline.org), suggesting that the rescue of physical barrier function was possibly due to the upregulation of this filaggrin-processing protease.

### Ctsh-deficient count, mast cell degranulation, and proinflammatory molecule expression

**Fig 6:**
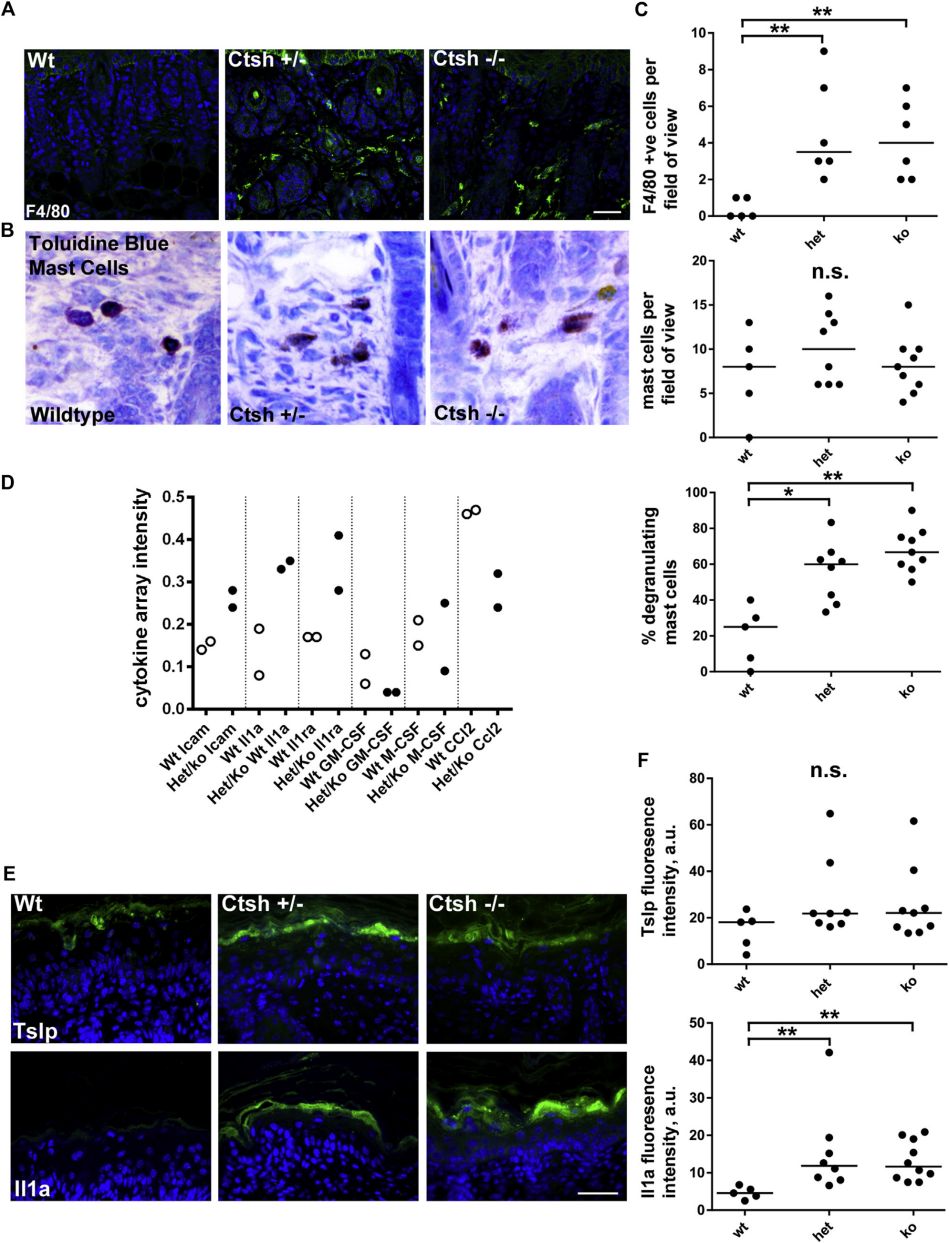
Loss of CTSH increases skin macrophage counts, mast cell degranulation, and proinflammatory molecule expression. **A,** Immunofluorescence of macrophages (F4/80^+^) in WT, *Ctsh*^*−/−*^*(ko)*, and *Ctsh*^*+/−*^*(het)* mouse neonatal skin. **B,** Toluidine blue staining. **C,** Graph of average F4/80^+^ cell and mast cell counts and percentage of degranulating mast cells per field of view. **D,** Densitometry of the cytokine arrays incubated with pooled lysates from 2 WT and 2 heterozygous or knockout mice (Het/Ko). **E,** Il1a and Tslp immunofluorescence. **F,** Graph of immunofluorescence intensity of Tslp and Il1a. *Bars* = 50 μm (Fig 6, *A*, *B*, and *E*). *P < .05 and **P < .005 (Fig 6, *C* and *F*). *n.s*., Not significant.

Defects in the physical barrier in patients with AD result in an immune response,^29^ which typically includes an increase in mast cell numbers and macrophage and lymphocyte infiltration.^30^, ^31^ We saw no change in CD45^+^ cell (lymphocytes) numbers in the dermis or epidermis of either the *Ctsh*^*+/−*^ or *Ctsh*^*−/−*^ mice (Fig 6, *A*, and see Fig E6, *B*). Increased macrophage numbers in the skin are associated with filaggrin-defective and barrier-defective epidermis.^32^, ^33^ Consistent with this, macrophage (F4/80^+^ cell) counts were increased in skin of the *Ctsh*^*+/−*^ and *Ctsh*^*−/−*^ mice (Fig 6, *A* and *C*). Mast cell degranulation and the release of histamine, proteases, and other immune mediators are common phenomena linked to the atopic phenotype,^34^ and although overall mast cell number was unchanged, degranulation was increased in the skin of the *Ctsh*^*+/−*^ and *Ctsh*^*−/−*^ mice (Fig 6, *B* and *C*).

**Fig 7:**
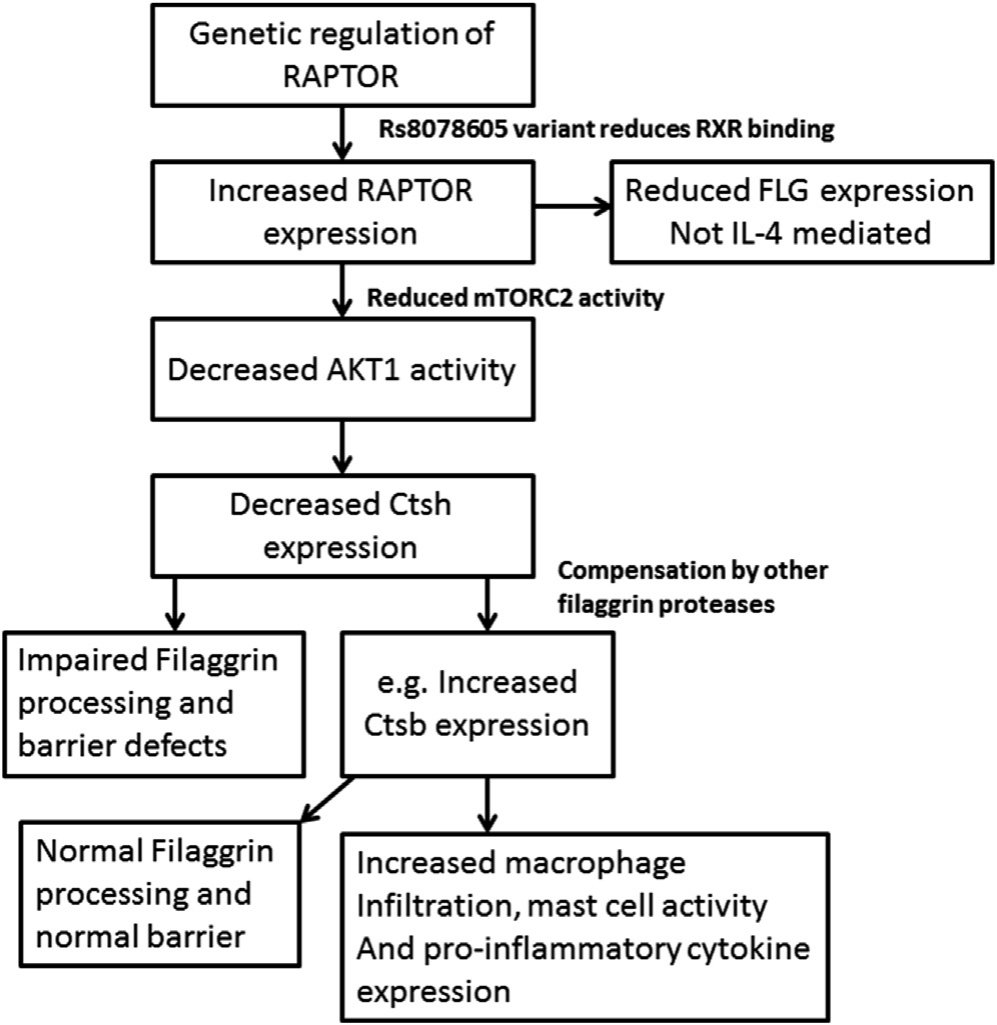
The mTORC/AKT1/CTSH axis in control of the physical and immune skin barrier. The variant SNP rs8078605 prevents RXR binding to the putative intragenic enhancer in RAPTOR, potentially increasing RAPTOR expression, which itself reduces filaggrin expression. This increases the ratio of mTORC1 to mTORC2, reducing Akt1 phosphorylation. This leads to reduced CTSH expression and decreases filaggrin processing. Upregulation of other filaggrin-processing proteases in response, such as cathepsin B, not only leads to rescue of barrier function but also causes macrophage infiltration, mast cell activity, and proinflammatory cytokine expression.

To determine whether the skin was more proinflammatory, we investigated cytokine and related protein expression using antibody array dot blots in pooled lysates from whole skin from WT and polled *Ctsh*^*−/−*^ and *Ctsh*^*+/−*^ newborn mouse skin (Fig 6, *D*). There was an increase in the expression of a number of cytokines and soluble immune mediators, including IL-1a, a protein with levels known to be increased in barrier-defective skin and skin of subjects with eczema,^35^ which was subsequently confirmed by means of immunofluorescence (Fig 6, *D-F*). Tslp expression induces AD in mouse models and is present in lesional AD skin.^36^ It also plays a key role in mast cell degranulation. However, we saw no significant change in Tslp expression in the epidermis of *Ctsh*^*−/−*^ and *Ctsh*^*+/−*^ newborn and adult mouse skin (Fig 6, *E* and *F*). Taken together, these data suggested that loss of Ctsh mediated by RAPTOR increase and AKT1 activity loss in patients with AD leads to mild epidermal barrier disruption and that the epidermis subsequently becomes more proinflammatory. In addition, although some aspects of the physical barrier are rescued in the knockout mouse, potentially because of compensation by cathepsin B and increased loricrin expression, the immune phenotype is not rescued (Fig 7).

## Discussion

Although there has been a great deal of study of *FLG* mutations and their association with barrier disruption and AD, there are surprisingly few reports on variation of filaggrin protein levels and filaggrin processing.^37^, ^38^, ^39^ Here we show that the increase in RAPTOR expression correlates with the decrease in filaggrin expression and processing not only in patients with AD but also in healthy “unaffected” subjects. This is consistent with other work on filaggrin proteases in patients with AD.^13^ Taken together, these data strongly suggest that there would be value in assessing genetic variants in the healthy population as a whole that correlate to barrier disruption and filaggrin expression and processing and disregarding AD because this might be a downstream consequence of the silent barrier disruption that is potentially mediated by its own set of genetic associations.^25^, ^40^, ^41^

Our analysis suggested that retinoids could be used as a treatment to reduce RAPTOR expression in patients with AD and hence increase filaggrin expression and processing. Retinoids have been used to successfully treat eczema in a number of studies.^42^, ^43^, ^44^ Typically, around 50% of patients respond to retinoid treatment.^42^ Although the immunosuppressive properties of retinoids are cited as the cause of recovery, another reason could be the reduction of RAPTOR expression and subsequent increase in filaggrin expression and processing. Both CTSH and filaggrin have been reported previously as being upregulated by retinoids, which is consistent with this hypothesis.^43^, ^45^, ^46^ It would be interesting to investigate epidermal RAPTOR, CTSH, and filaggrin levels and processing before and after treatment with retinoids and to determine whether there is a different response in patients with different FLG phenotypes. A potential complication would be that treatment with all-trans retinoic acid or retinoic acid metabolism inhibitors can both inhibit and enhance epidermal terminal differentiation,^47^, ^48^, ^49^, ^50^ and therefore the potential overall effect on epidermal barrier function would be hard to predict.

Interestingly, in the context of the skin barrier and RAPTOR, mTORC1 is a pH sensor, and at acidic pH, such as that encountered in the granular layer of the epidermis, mTORC1 is inhibited.^51^ This should decrease filaggrin expression and would be balanced against filaggrin-derived urocanic acid and pyrrolidone carboxylic acid levels.^52^ Coupled with the fact that CTSH is a lysosomal protease and therefore active at acidic pHs, it is likely that pH is one of the factors that determine overall levels of processed filaggrin.

The skin of Akt1 null mice models and Akt1 knockdown organotypic cultures display hyperkeratosis with reduced cornified envelope strength and reduced filaggrin expression and processing.^17^, ^53^, ^54^ Activation of Akt1 also results in hyperkeratosis and altered filaggrin expression,^17^, ^54^ demonstrating that normal Akt activity levels are required for correct filaggrin processing and hence epidermal barrier function. The new findings presented here reveal CTSH to be required for filaggrin processing and epidermal barrier formation and that in the skin RAPTOR regulates CTSH expression and filaggrin processing through reduced Akt signaling.

CTSH is expressed ubiquitously, and in addition to being involved in bulk protein degradation, it does display cell-specific functions, such as its role in the processing and secretion of surfactant protein C in type II pneumocytes.^19^, ^55^ Ctsh-deficient mice have reduced lung surfactant levels, which might interfere with breathing mechanisms, causing respiratory complications.^19^ Furthermore, reduced *Ctsh* mRNA expression in airway smooth muscle cells has been reported in asthmatic patients,^56^ suggesting the possibility that low levels of Akt signaling might, in a range of epithelia, contribute to progression of AD to other atopic disease, the so-called atopic march.^57^ The finding that Ctsh is either directly or indirectly involved through the activation of other proteases, such as granzymes,^57^ in the processing of key barrier proteins in the epidermis and in the lung leads to the possibility that the atopic march might not only be an immunologic phenomenon but could also be the result of altered barrier function in multiple epithelia.

CTSH deficiency *in vivo* led to an increase in macrophage numbers and mast cell degranulation and increased IL-1a levels in the skin of *Ctsh*^*+/−*^ and *Ctsh*^*−/−*^ mice. CTSH overexpression typically correlates to macrophage infiltration and a proinflammatory environment in a number of tissues.^58^, ^59^ Therefore it is likely that the loss of Ctsh leads to an increase in levels of other cathepsins, such as our observation of increased levels of cathepsin B, which might have a proinflammatory role and is known to play an important role in processing of mast cell proteases.^60^, ^61^ Therefore it is possible that the immune changes are driven by increased cathepsin B levels in both *Ctsh*^*+/−*^ and *Ctsh*^*−/−*^ mice. The interplay between these proteases and inhibitors and how this relates to levels of filaggrin and other related (fused-S100 group) proteins and their processing and subsequently the proinflammatory status of the skin in patients with AD is difficult to dissect. This was apparent by the lack of correlation between filaggrin levels and CTSH in patients with AD. However, understanding how overall filaggrin protease activity levels are altered in atopic skin would provide targets to treat both the barrier and immune aspects of AD.

Subjects with 2 loss-of-function mutations in *FLG* (compound heterozygotes) show the greatest increase in risk of AD,^62^, ^63^ and gene expression differences in these subjects is greater than in *FLG* heterozygote and WT subjects,^21^ which allowed for the detection of statistically significant differentially expressed genes correlated with RAPTOR expression. Consistent with our work *in vitro*, high levels of RAPTOR correlated with low levels of filaggrin expression and AKT signaling components. Taken together, our findings make a convincing case for the role of RAPTOR in regulating genes, including *FLG*, that are important in the AD phenotype. Also, this work suggests that rapamycin or retinoid treatment could be of benefit in these patients with filaggrin haploinsufficiency and severe AD.

**Key messages**

• RAPTOR expression is increased in patients with AD and are inversely proportional to filaggrin expression.
• The upregulation of RAPTOR leads to AKT1 activity downregulation and downregulation of the protease CTSH, which is involved in filaggrin processing and epidermal barrier function and modulates skin immunity.

## Supplementary data

Online Repository text

Tables E1-E4
