# Supplementary material for: A mechanistic target of rapamycin complex 1/2 (mTORC1)/V-Akt murine thymoma viral oncogene homolog 1 (AKT1)/cathepsin H axis controls filaggrin expression and processing in skin, a novel mechanism for skin barrier disruption in patients with atopic dermatitis
Source: J Allergy Clin Immunol. 2017 Apr;139(4):1228–41. doi: 10.1016/j.jaci.2016.09.052 (PMC5380661; doi:10.1016/j.jaci.2016.09.052)
Supplement: Online Repository text [file mmc1.docx]

**Supplementary Figure E1: Raptor and Filaggrin in normal skin and non lesional AD skin; IL-4 expression (a)** Left, densitometry of filaggrin in Normal and AD non-lesional skin. Middle, Filaggrin levels in high and low RAPTOR expressing non-lesional AD patient skin. Right, Filaggrin levels in high and low RAPTOR expressing normal skin. *p<0.05, ** p<0.005 Mann-Whitney U-test. Bars, left and middle are the interquartile range. **(b)** IL-4 Western blot in rat epidermal keratinocytes treated with rapamycin (Rapa) for 24 hours (10nM). **(c)** IL-4 Western blot in AKT1 kd human keratinocytes. (b and c). Gapdh is loading control in (b,c).

**Supplementary Figure E2: Analysis of the highly differentially expressed genes in AD which anti-correlate and correlate with RAPTOR expression. (a)** STRING (<http://string-db.org/>) network of functionally interacting genes, with RAPTOR and AKT1 and the anti-correlated genes in green and red for anti-correlated and correlated genes respectively. Highly expressed genes that also correlated with FLG expression are indicated with an asterisk **(b)** Venn diagram showing the large overlap betweenhighly expressed genes correlating with RAPTOR expression and genes previously determined^14^ to be correlated with the loss of filaggrin expression.

**Supplementary figure E3: a SNP variant correlates with increased RAPTOR expression, reduced filaggrin expression and processing and reduced Cathepsin H expression. (a)** Genomic context and of SNP rs8078605 and a graph of the GWAS data^63^ from the RAPTOR region, y-axis, LOD score, the p-value for rs8078605 was 0.067. **(b)** Piechart showing prevalence of each genotype of the SNP rs8078605 in European and sub-Saharan African populations, Normal and AD individuals **(c)** RAPTOR expression in human keratinocytes in response to ATRA. Gapdh is loading control. Bar chart shows RAPTOR densitometry in 2 separate experiments **(d)** RAPTOR densitometry of western blots of 9 human skin samples with the C/ C (n=6), T/C (n=2) or T/T (n=1) variants in rs8078605. **(e)** Plot of RAPTOR densitometry against normalised western blot densitometry of a corresponding filaggrin western blot. T/T and C/T rs8078705 variants are marked on the graph, as is the correlation coefficient (R^2^). **(f)** filaggrin and Ctsh Western blots from human samples, keratin 5 is an epidermal loading control.

**Supplementary figure E4: Analysis of genes differentially expressed in Akt1kd keratinocytes. (a)** Graph of enrichment scores for all significantly differentially expressed genes, including Ctsh **(b)** Graph of enrichment scores of genes involved in MTORC signalling **(c)**. Graph of 1/p values (uncorrected) of the three most over-represented functional groups in scrambled control cells by GSEA analysis. **(d)**. Leading edge analysis of the most differentially expressed genes in these three ontology groups, with several genes including HMGCS1 present in all gene ontology groups. **(e)** HMGCS1 expression in AD according to the RNAseq data in Cole et al., 2013.

**Supplementary figure E5: Cathepsin H is required for Filaggrin processing but expression does not correlate with Filaggrin in atopic dermatitis. (a)** Western blot of filaggrin and Cathepsin H in 4 Ctsh kd lines. **(b)** Graph of mean densitometry of total filaggrin, filaggrin monomer and Ctsh, n=4 **(c)** Real time PCR analysis of filaggrin expression in two Ctsh shRNA lines. **(d**) cathepsin H expression represented from RNAseq analysis in Cole et al., 2014. Box shows median and interquartile ranges in wildtype controls and the three eczema *FLG* phenotypes **(e)** Scatterplots showing Pearson correlation (x-axis) of gene expression levels with cathepsin H expression. The fold-change of all significantly differentially expressed genes (FDR p< 0.05) are represented on the y axis, with Filaggrin (FLG) in orange. Correlations are between FLG wildtype, FLG heterozygous and FLG compound heterozygous (n=7).

**Supplementary figure E6: Cathepsin B expression increases in Cathepsin H deficient mouse epidermis. (a)** Ctsh and Cathepsin B (Ctsb) immunofluoresence in Ctsh -/-, Ctsh +/- and wt mouse epidermis. Graph shows Ctsb intensity in the neonate epidermis, bars are median **(b)** CD45 Immunofluorescence and in the dermis of Ctsh -/-, Ctsh +/- , and wt mouse skin. *p<0.05, **p<0.005 Bar 50µm.

**Supplementary Figure E7 – Barrier proteins and immune mediators in adult Ctsh +/- and -/- mouse epidermis(a)** histology, filaggrin and loricrin immunofluoresence of adult mouse Ctsh +/-, -/- and wt epidermis. **(b)** Il1a and Tslp immunofluorescence of Il1a and Tslp adult mouse Ctsh +/-, -/- and wt epidermis **(c)** Western blot of filaggrin, keratin 10 and loricrin **(d)** Graphs of densitometry of total filaggrin and filaggrin monomer. p values are shown on the graph. bars 50 µm (a,b)

**Supplementary Methods**

**Restriction fragment length polymorphism analysis**

RFLP analysis was performed on 18 skin samples. DNA was extracted by DNA mini spin kit (Qiagen) according to manufacturers’ instructions. The rs8078605 polymorphism introduced a BsmAI site into the locus. F- CACCGCATTTGCTCTTACAA and R- CCTACACATGGTCCTTCATCC (Tm 60^o^C) primers produced a 454bp amplicon. The T variant after BsmAI digestion gives a 203bp and 251bp product.
