# Supplementary material for: A mechanistic target of rapamycin complex 1/2 (mTORC1)/V-Akt murine thymoma viral oncogene homolog 1 (AKT1)/cathepsin H axis controls filaggrin expression and processing in skin, a novel mechanism for skin barrier disruption in patients with atopic dermatitis
Source: J Allergy Clin Immunol. 2017 Apr;139(4):1228–41. doi: 10.1016/j.jaci.2016.09.052 (PMC5380661; doi:10.1016/j.jaci.2016.09.052)
Supplement: Tables E1-E4 [file mmc2.docx]

**Supplementary table E1**

**Patient information for the 5 AD patients from Great Ormond Street Hospital.** Location of biopsy for the non-lesional samples. Previous treatment, AZA, azathioprine; CSA cyclosporine

| **Patient** | **Sex** | **Age** | **Location of Biopsy** | **Previous Treatment** |
| --- | --- | --- | --- | --- |
| 1 | F | 13 | R Waist | oral steroid and AZA |
| 2 | M | 13 | L Arm | AZA |
| 3 | M | 13 | L Thigh | AZA |
| 4 | F | 12 | R Upper leg | AZA and CSA |
| 5 | M | 11 | Lower Back | No systemic treatment |

**Patient information for the Cole et al, cohort^14^:** biopsies were taken from the non-lesional skin (the upper buttock with no clinical signs of active inflammation) of children aged 6 to 16 years who had early onset, persistent and severe atopic eczema. The 10 controls were non-atopic individuals ie no eczema, asthma or hay fever. Severity measurement is by physician global assessment

|  | **severity at time of biopsy** |  |  |  |  |
| --- | --- | --- | --- | --- | --- |
|  | **mild** | **moderate** | **severe** | **not recorded** | **total** |
| **FLG wt** | 1 | 4 | 2 | 0 | 7 |
| **FLG het** | 3 | 4 | 4 | 1 | 12 |
| **FLG compound het** | 1 | 2 | 3 | 1 | 7 |
| **Total** | 5 | 10 | 9 | 2 | 26 |

**Supplementary table E2:** List of the 84 genes strongly correlated or anti-correlated with RAPTOR expression levels in unaffected compound heterozygote AD patient skin that are significantly differentially expressed in Cole et al 2014^14^, GeneName, official HUGO nomenclature, WT, Het and Cmpd, are mean normalised expression levels of WT, Heterozygote and Compound Heterozygote respectively, s.d are the standard deviations of each cohort. Cor, Pearson correlation coefficient. FC fold change, pval is the p value after correction for multiple testing

| Gene | Description | WT | Het | Cmpd | WT.sd | Het.sd | Cmpd.sd | cor | FC | logFC | pval |
| --- | --- | --- | --- | --- | --- | --- | --- | --- | --- | --- | --- |
| PTPRC | protein tyrosine phosphatase, receptor type, C | 6.6 | 11 | 16 | 3 | 14 | 21 | 1 | 2.5 | 1.3 | 0.0079 |
| IRF1 | interferon regulatory factor 1 | 18 | 30 | 43 | 6.9 | 44 | 73 | 1 | 2.4 | 1.3 | 0.012 |
| ISG15 | ISG15 ubiquitin-like modifier | 12 | 19 | 27 | 5.1 | 35 | 45 | 1 | 2.3 | 1.2 | 0.023 |
| HAPLN3 | hyaluronan and proteoglycan link protein 3 | 7.2 | 11 | 14 | 2 | 12 | 19 | 1 | 1.9 | 0.91 | 0.023 |
| TIMM22 | translocase of inner mitochondrial membrane 22 homolog (yeast) | 9.6 | 13 | 17 | 3.6 | 6.5 | 11 | 1 | 1.8 | 0.84 | 0.0074 |
| CKAP2 | cytoskeleton associated protein 2 | 15 | 20 | 26 | 2.9 | 7.3 | 6.5 | 1 | 1.7 | 0.78 | 0.0013 |
| CARD10 | caspase recruitment domain family, member 10 | 12 | 16 | 21 | 5.6 | 5.6 | 8.1 | 1 | 1.7 | 0.78 | 0.014 |
| TAP2 | transporter 2, ATP-binding cassette, sub-family B (MDR/TAP) | 38 | 47 | 62 | 7.9 | 33 | 61 | 1 | 1.7 | 0.73 | 0.031 |
| TRIM22 | tripartite motif containing 22 | 28 | 39 | 47 | 4.7 | 52 | 63 | 0.99 | 1.7 | 0.76 | 0.049 |
| DBF4 | DBF4 zinc finger | 8 | 10 | 13 | 3.3 | 2.7 | 2.4 | 1 | 1.6 | 0.7 | 0.0071 |
| UBE2L6 | ubiquitin-conjugating enzyme E2L 6 | 25 | 32 | 38 | 8.6 | 24 | 37 | 1 | 1.5 | 0.59 | 0.0085 |
| SNX11 | sorting nexin 11 | 8.3 | 10 | 12 | 1.5 | 2.5 | 2.9 | 1 | 1.5 | 0.55 | 0.009 |
| GXYLT1 | glucoside xylosyltransferase 1 | 7.2 | 8.6 | 10 | 2 | 2.2 | 3.2 | 1 | 1.5 | 0.54 | 0.0096 |
| IFI27 | interferon, alpha-inducible protein 27 | 190 | 230 | 280 | 140 | 340 | 450 | 1 | 1.5 | 0.54 | 0.011 |
| PSMB10 | proteasome (prosome, macropain) subunit, beta type, 10 | 13 | 16 | 19 | 4.2 | 12 | 17 | 1 | 1.5 | 0.57 | 0.015 |
| LCP2 | lymphocyte cytosolic protein 2 (SH2 domain containing leukocyte protein of 76kDa) | 8.2 | 9.8 | 12 | 2.1 | 8.1 | 8.3 | 1 | 1.5 | 0.56 | 0.023 |
| PARP9 | poly (ADP-ribose) polymerase family, member 9 | 31 | 40 | 48 | 12 | 46 | 54 | 1 | 1.5 | 0.62 | 0.024 |
| NUAK2 | NUAK family, SNF1-like kinase, 2 | 18 | 23 | 27 | 11 | 12 | 13 | 1 | 1.5 | 0.55 | 0.026 |
| KCNK1 | potassium channel, two pore domain subfamily K, member 1 | 30 | 37 | 44 | 7 | 9.2 | 6.7 | 0.99 | 1.5 | 0.55 | 0.035 |
| HLA-DOA | major histocompatibility complex, class II, DO alpha | 13 | 15 | 18 | 3.4 | 6.9 | 13 | 1 | 1.4 | 0.51 | 0.012 |
| PRAF2 | PRA1 domain family, member 2 | 19 | 16 | 14 | 4.3 | 3.4 | 4.4 | -1 | 0.71 | -0.5 | 0.025 |
| NOV | nephroblastoma overexpressed | 53 | 46 | 37 | 16 | 14 | 8.9 | -1 | 0.7 | -0.51 | 0.0049 |
| ZBTB14 | zinc finger and BTB domain containing 14 | 13 | 11 | 9 | 3.6 | 2.8 | 2.2 | -1 | 0.7 | -0.52 | 0.017 |
| ENTPD4 | ectonucleoside triphosphate diphosphohydrolase 4 | 18 | 15 | 12 | 3.6 | 3 | 2.9 | -1 | 0.7 | -0.52 | 0.022 |
| SLC9B2 | solute carrier family 9, subfamily B (NHA2, cation proton antiporter 2), member 2 | 17 | 15 | 12 | 5.2 | 3.7 | 3.1 | -1 | 0.7 | -0.51 | 0.026 |
| TPM2 | tropomyosin 2 (beta) | 110 | 90 | 75 | 66 | 40 | 21 | -1 | 0.7 | -0.52 | 0.028 |
| LAMA3 | laminin, alpha 3 | 19 | 16 | 13 | 9.2 | 3.8 | 5.1 | -1 | 0.7 | -0.52 | 0.041 |
| RHOU | ras homolog family member U | 17 | 14 | 12 | 7.4 | 5.8 | 3.5 | -1 | 0.7 | -0.51 | 0.046 |
| ABHD4 | abhydrolase domain containing 4 | 11 | 9.8 | 7.9 | 1.7 | 2.3 | 1.7 | -1 | 0.69 | -0.54 | 0.0028 |
| MXRA8 | matrix-remodelling associated 8 | 21 | 18 | 15 | 8.3 | 5.4 | 4.7 | -1 | 0.69 | -0.54 | 0.01 |
| CD1A | CD1a molecule | 31 | 26 | 21 | 12 | 8.7 | 9.5 | -1 | 0.69 | -0.53 | 0.021 |
| RNF152 | ring finger protein 152 | 43 | 36 | 30 | 7.6 | 8.9 | 6.8 | -1 | 0.69 | -0.53 | 0.024 |
| CLDN10 | claudin 10 | 13 | 11 | 8.7 | 9.5 | 5.7 | 5.5 | -1 | 0.68 | -0.56 | 0.032 |
| GPR137 | G protein-coupled receptor 137 | 11 | 8.9 | 7.4 | 3.6 | 2.3 | 3 | -1 | 0.68 | -0.55 | 0.04 |
| ZDHHC11 | zinc finger, DHHC-type containing 11 | 29 | 24 | 20 | 16 | 12 | 12 | -1 | 0.68 | -0.56 | 0.041 |
| RP11-613D13.4 | none | 28 | 25 | 19 | 17 | 8 | 6.6 | -1 | 0.68 | -0.56 | 0.045 |
| LPCAT1 | lysophosphatidylcholine acyltransferase 1 | 15 | 12 | 10 | 4.2 | 3.8 | 3.2 | -1 | 0.68 | -0.56 | 0.046 |
| DCLK1 | doublecortin-like kinase 1 | 18 | 16 | 12 | 9.7 | 7.1 | 7 | -1 | 0.68 | -0.55 | 0.049 |
| MT-ND1 | mitochondrially encoded NADH dehydrogenase 1 | 360 | 310 | 240 | 92 | 91 | 40 | -1 | 0.67 | -0.57 | 0.0023 |
| NCALD | neurocalcin delta | 38 | 33 | 25 | 16 | 12 | 10 | -1 | 0.67 | -0.58 | 0.017 |
| NNMT | nicotinamide N-methyltransferase | 30 | 26 | 20 | 13 | 14 | 6.6 | -1 | 0.67 | -0.57 | 0.017 |
| WNK2 | WNK lysine deficient protein kinase 2 | 12 | 11 | 8.4 | 3.8 | 4 | 4 | -1 | 0.67 | -0.57 | 0.019 |
| PIGV | phosphatidylinositol glycan anchor biosynthesis, class V | 11 | 9.6 | 7.4 | 1.7 | 3.5 | 2.9 | -1 | 0.67 | -0.58 | 0.022 |
| CCNG2 | cyclin G2 | 27 | 24 | 18 | 11 | 8.1 | 11 | -1 | 0.67 | -0.58 | 0.036 |
| PRELP | proline/arginine-rich end leucine-rich repeat protein | 60 | 49 | 40 | 23 | 8.1 | 11 | -1 | 0.67 | -0.57 | 0.041 |
| C11orf96 | chromosome 11 open reading frame 96 | 28 | 25 | 19 | 17 | 8 | 6 | -1 | 0.67 | -0.58 | 0.049 |
| FLNC | filamin C, gamma | 13 | 11 | 8.3 | 5.9 | 3.8 | 4 | -1 | 0.66 | -0.59 | 0.0016 |
| SNED1 | sushi, nidogen and EGF-like domains 1 | 11 | 9.8 | 7.6 | 5.5 | 4.2 | 3.9 | -1 | 0.66 | -0.59 | 0.0072 |
| FMOD | fibromodulin | 32 | 28 | 21 | 13 | 9.1 | 6.7 | -1 | 0.66 | -0.6 | 0.028 |
| MT-ND5 | mitochondrially encoded NADH dehydrogenase 5 | 630 | 550 | 410 | 300 | 240 | 180 | -1 | 0.66 | -0.61 | 0.035 |
| TLE2 | transducin-like enhancer of split 2 | 13 | 12 | 8.6 | 4.3 | 3 | 3.5 | -1 | 0.66 | -0.6 | 0.049 |
| UTY | ubiquitously transcribed tetratricopeptide repeat containing, Y-linked | 13 | 11 | 8.6 | 5.2 | 6.9 | 5.4 | -1 | 0.65 | -0.62 | 0.015 |
| CYBA | cytochrome b-245, alpha polypeptide | 11 | 9.3 | 7.3 | 3.3 | 4.8 | 7.1 | -1 | 0.65 | -0.63 | 0.021 |
| KLF9 | Kruppel-like factor 9 | 60 | 49 | 39 | 36 | 18 | 12 | -1 | 0.64 | -0.65 | 0.023 |
| ZDHHC11B | zinc finger, DHHC-type containing 11B | 26 | 23 | 17 | 17 | 12 | 8.3 | -1 | 0.64 | -0.64 | 0.032 |
| THBS1 | thrombospondin 1 | 32 | 25 | 20 | 19 | 9 | 6.6 | -1 | 0.64 | -0.65 | 0.039 |
| CRELD1 | cysteine-rich with EGF-like domains 1 | 22 | 17 | 14 | 6.7 | 3 | 4.7 | -1 | 0.64 | -0.65 | 0.046 |
| RAI2 | retinoic acid induced 2 | 15 | 13 | 9.6 | 3.9 | 4.7 | 4.6 | -1 | 0.63 | -0.68 | 0.021 |
| NOVA1 | neuro-oncological ventral antigen 1 | 20 | 17 | 12 | 5 | 5.6 | 6.2 | -1 | 0.63 | -0.67 | 0.046 |
| EBF1 | early B-cell factor 1 | 19 | 16 | 12 | 8.8 | 6 | 3.3 | -1 | 0.62 | -0.68 | 0.014 |
| FAM13A | family with sequence similarity 13, member A | 39 | 31 | 24 | 33 | 11 | 5.1 | -1 | 0.62 | -0.7 | 0.014 |
| HNMT | histamine N-methyltransferase | 19 | 15 | 12 | 4.3 | 5 | 5.2 | -1 | 0.62 | -0.68 | 0.036 |
| ZG16B | zymogen granule protein 16B | 49 | 38 | 30 | 32 | 15 | 12 | -1 | 0.62 | -0.69 | 0.043 |
| IGF2 | insulin-like growth factor 2 | 21 | 17 | 13 | 12 | 6.7 | 3.8 | -1 | 0.61 | -0.7 | 0.0099 |
| MT-CO1 | mitochondrially encoded cytochrome c oxidase I | 460 | 370 | 280 | 120 | 140 | 59 | -1 | 0.61 | -0.71 | 0.016 |
| HOTAIR | HOX transcript antisense RNA | 13 | 11 | 7.8 | 7.4 | 3.3 | 5.4 | -1 | 0.6 | -0.73 | 0.0056 |
| MXRA7 | matrix-remodelling associated 7 | 15 | 12 | 9.1 | 5.4 | 3.2 | 2.7 | -1 | 0.6 | -0.74 | 0.013 |
| INSR | insulin receptor | 14 | 12 | 8.7 | 7.8 | 3.4 | 5.3 | -1 | 0.6 | -0.73 | 0.024 |
| LIG1 | ligase I, DNA, ATP-dependent | 15 | 12 | 8.7 | 3.1 | 5.3 | 3.5 | -1 | 0.6 | -0.75 | 0.031 |
| SPRN | shadow of prion protein homolog (zebrafish) | 14 | 11 | 8.2 | 5.3 | 6.1 | 4.6 | -1 | 0.58 | -0.79 | 0.027 |
| HRH1 | histamine receptor H1 | 11 | 9.4 | 6.3 | 5.6 | 4.7 | 3.4 | -1 | 0.58 | -0.78 | 0.047 |
| RGCC | regulator of cell cycle | 74 | 58 | 42 | 29 | 25 | 11 | -1 | 0.57 | -0.8 | 0.012 |
| PRR4 | proline rich 4 (lacrimal) | 54 | 40 | 30 | 60 | 69 | 24 | -1 | 0.56 | -0.83 | 0.05 |
| S100P | S100 calcium binding protein P | 62 | 47 | 34 | 18 | 17 | 24 | -1 | 0.54 | -0.89 | 0.026 |
| IGFBP6 | insulin-like growth factor binding protein 6 | 84 | 65 | 45 | 40 | 21 | 8.3 | -1 | 0.53 | -0.91 | 0.0075 |
| MUCL1 | mucin-like 1 | 460 | 350 | 240 | 290 | 190 | 200 | -1 | 0.53 | -0.92 | 0.028 |
| KIAA1841 | KIAA1841 | 21 | 17 | 11 | 8.3 | 4.9 | 3.6 | -1 | 0.5 | -1 | 0.017 |
| MT-CO2 | mitochondrially encoded cytochrome c oxidase II | 110 | 89 | 54 | 36 | 53 | 19 | -1 | 0.5 | -0.99 | 0.043 |
| C2orf74 | chromosome 2 open reading frame 74 | 19 | 15 | 8.5 | 8.2 | 5.2 | 3.1 | -1 | 0.46 | -1.1 | 0.044 |
| HSPB6 | heat shock protein, alpha-crystallin-related, B6 | 24 | 19 | 10 | 14 | 7.6 | 6.8 | -1 | 0.43 | -1.2 | 0.04 |
| CYP4B1 | cytochrome P450, family 4, subfamily B, polypeptide 1 | 22 | 14 | 8.6 | 13 | 6.8 | 4.2 | -1 | 0.4 | -1.3 | 0.042 |
| CILP | cartilage intermediate layer protein, nucleotide pyrophosphohydrolase | 33 | 25 | 11 | 18 | 19 | 7.9 | -1 | 0.32 | -1.6 | 0.032 |
| FLG | filaggrin | 3300 | 1900 | 920 | 680 | 460 | 270 | -1 | 0.28 | -1.8 | 0.044 |
| SCGB1D2 | secretoglobin, family 1D, member 2 | 110 | 61 | 30 | 63 | 65 | 17 | -1 | 0.28 | -1.8 | 0.049 |

**Supplementary Table E3** Concordance of the top 22 highly expressed and differentially expressed genes strongly correlated or anti-correlated with RAPTOR expression with gene whose expression level correlated with FLG expression levels^14^ in unaffected compound heterozygote AD patient skin that are significantly differentially expressed, Gray denote either positive or negative correlation in both analyses. GeneName, official HUGO nomenclature, WT, Het and Cmpd, are mean normalised expression levels of WT, Heterozygote and Compound Heterozygote respectively, s.d are the standard deviations of each cohort. Cor, pearson correlation coefficient. FC fold change, pval is the p value after correction for multiple testing

| **Gene** | **Description** | **WT** | **Cmpd** | **FC** | **logFC** | **pval** |
| --- | --- | --- | --- | --- | --- | --- |
| **TAP2** | **transporter 2, ATP-binding cassette, sub-family B (MDR/TAP)** | 38 | 62 | 1.7 | 0.73 | **0.031** |
| **TRIM22** | **tripartite motif containing 22** | 28 | 47 | 1.7 | 0.76 | **0.049** |
| **IFI27** | **interferon, alpha-inducible protein 27** | 190 | 280 | 1.5 | 0.54 | **0.011** |
| **KCNK1** | **potassium channel, two pore domain subfamily K, member 1** | 30 | 44 | 1.5 | 0.55 | **0.035** |
| **PARP9** | **poly (ADP-ribose) polymerase family, member 9** | 31 | 48 | 1.5 | 0.62 | **0.024** |
| **NOV** | **nephroblastoma overexpressed** | 53 | 37 | 0.7 | -0.51 | **0.005** |
| **TPM2** | **tropomyosin 2 (beta)** | 110 | 75 | 0.7 | -0.52 | **0.028** |
| **RNF152** | **ring finger protein 152** | 43 | 30 | 0.7 | -0.53 | **0.024** |
| **MT-ND1** | **mitochondrially encoded NADH dehydrogenase 1** | 360 | 240 | 0.7 | -0.57 | **0.002** |
| **PRELP** | **proline/arginine-rich end leucine-rich repeat protein** | 60 | 40 | 0.7 | -0.57 | **0.041** |
| **MT-ND5** | **mitochondrially encoded NADH dehydrogenase 5** | 630 | 410 | 0.7 | -0.61 | **0.035** |
| **KLF9** | **Kruppel-like factor 9** | 60 | 39 | 0.6 | -0.65 | **0.023** |
| **ZG16B** | **zymogen granule protein 16B** | 49 | 30 | 0.6 | -0.69 | **0.043** |
| **MT-CO1** | **mitochondrially encoded cytochrome c oxidase I** | 460 | 280 | 0.6 | -0.71 | **0.016** |
| **RGCC** | **regulator of cell cycle** | 74 | 42 | 0.6 | -0.8 | **0.012** |
| **PRR4** | **proline rich 4 (lacrimal)** | 54 | 30 | 0.6 | -0.83 | **0.05** |
| **S100P** | **S100 calcium binding protein P** | 62 | 34 | 0.5 | -0.89 | **0.026** |
| **IGFBP6** | **insulin-like growth factor binding protein 6** | 84 | 45 | 0.5 | -0.91 | **0.008** |
| **MUCL1** | **mucin-like 1** | 460 | 240 | 0.5 | -0.92 | **0.028** |
| **MT-CO2** | **mitochondrially encoded cytochrome c oxidase II** | 110 | 54 | 0.5 | -0.99 | **0.043** |
| **FLG** | **filaggrin** | 3300 | 920 | 0.3 | -1.8 | **0.044** |
| **SCGB1D2** | **secretoglobin, family 1D, member 2** | 110 | 30 | 0.3 | -1.8 | **0.049** |

**Supplementary Table E4**

A table showing the average fold change in expression in both Akt1 kd lines of all genes 2-fold and above differentially expressed; The 1.5 –fold or more down-regulated genes related to MTORC signalling and Proteases in the GSEA analysis are also shown in this table.

| 2-fold up- and down-regulated genes | |  |
| --- | --- | --- |
| **Symbol** | **Entrez Gene Name** | **Fold Change** |
| Khdrbs3 | KH domain containing, RNA binding, signal transduction associated 3 | 7.4 |
| Pdlim2 | PDZ and LIM domain 2 | 7.2 |
| Ckmt1 | creatine kinase, mitochondrial 1 | 5.2 |
| Tmbim4 | transmembrane BAX inhibitor motif containing 4 | 5.1 |
| Bin3 | bridging integrator 3 | 5.1 |
| Sema3a | sema domain, immunoglobulin domain (Ig), short basic domain, secreted, (semaphorin) 3A | 4.8 |
| Ppp3cc | protein phosphatase 3, catalytic subunit, gamma isoform | 4.7 |
| Cldn3 | claudin 3 | 3.7 |
| Asrgl1 | asparaginase like 1 | 3.6 |
| Ccbl1 | cysteine conjugate-beta lyase, cytoplasmic | 3.6 |
| Expi | extracellular proteinase inhibitor | 3.5 |
| Sepp1 | selenoprotein P, plasma, 1 | -2.1 |
| Fads1 | fatty acid desaturase 1 | -2.1 |
| Pkib | protein kinase (cAMP-dependent, catalytic) inhibitor beta | -2.1 |
| Il33 | interleukin 33 | -2.1 |
| Nt5e | 5' nucleotidase, ecto | -2.4 |
| Calml3 | calmodulin-like 3 | -2.7 |
| S100g | S100 calcium binding protein G | -2.7 |
| Slfn3 | schlafen 3 | -2.9 |
| Ctsh | cathepsin H | -3.9 |
| 1.5-fold or more down-regulated genes involved in mTORC signalling | | |
| Fads2 | Fatty Acid Desaturase 2 | -1.6 |
| Cth | Cystathionine Gamma-Lyase | -1.8 |
| Hmgcs1 | 3-Hydroxy-3-Methylglutaryl-CoA Synthase 1 (Soluble) | -1.9 |
| Elovl6 | ELOVL Fatty Acid Elongase 6 | -1.9 |
| Idi1 | Isopentenyl-Diphosphate Delta Isomerase 1 | -2.0 |
| Fads1 | Fatty Acid Desaturase 1 | -2.1 |
| 1.5-fold or more down-regulated proteases | | |
| Ace2 | Angiotensin I Converting Enzyme 2 | -1.6 |
| Pcsk6 | Proprotein Convertase Subtilisin/Kexin Type 6 | -1.6 |
| C1s | Complement Component 1, S Subcomponent | -1.6 |
| Ctsh | Cathepsin H | -3.9 |
